# Overexpression of angiogenic factors and matrix metalloproteinases in the saliva of oral squamous cell carcinoma patients: potential non-invasive diagnostic and therapeutic biomarkers

**DOI:** 10.1186/s12885-022-09630-0

**Published:** 2022-05-11

**Authors:** Meijuan Cai, Zhichao Zheng, Zhibao Bai, Kexiong Ouyang, Qiuyu Wu, Shaofen Xu, Lihuan Huang, Yingtong Jiang, Lijing Wang, Jie Gao, Janak L. Pathak, Lihong Wu

**Affiliations:** 1 Affiliated Stomatology Hospital of Guangzhou Medical University, Guangdong Engineering Research Center of Oral Restoration and Reconstruction, Guangzhou Key Laboratory of Basic and Applied Research of Oral Regenerative Medicine, Guangzhou, 510182 Guangdong China; 2 Stomatological Hospital, Southern Medical University, Guangzhou, 510280 Guangdong China; 3 Department of Basic Oral Medicine, Guangzhou Medical University School and Hospital of Stomatology, Guangzhou, 510182 Guangdong China; 4 Guangzhou First People’s Hospital, Guangzhou Medical University, Guangzhou, China; 5 Department of Oral and Maxillofacial Surgery, Guangzhou Key Laboratory of Basic and Applied Research of Oral Regenerative Medicine, Affiliated Stomatology Hospital of Guangzhou Medical University, Guangzhou, Guangdong 510182 China; 6 Department of Stomatology, Hexian Memorial Affiliated Hospital of Southern Medical University, Guangzhou, 511400 Guangdong China; 7 Vascular Biology Research Institute, Guangdong Pharmaceutical University, Guangzhou Higher Education Mega Center, Guangzhou, 510006 China

**Keywords:** Angiogenic factors, Diagnostic markers, MMPs, Oral squamous cell carcinoma, Saliva

## Abstract

**Backgrounds:**

Salivary biomarkers hold huge potential for the non-invasive diagnosis of oral squamous cell carcinoma. Angiogenic factors and matrix-metalloproteinases (MMPs) are highly expressed in OSCC tissue, but their expression patterns in the saliva are unknown. This study aimed to analyze the levels of angiogenic factors and MMPs in tumor tissue and saliva of OSCC patients.

**Methods:**

OSCC-tissue, adjacent normal tissue (ANT), saliva from OSCC patients, and healthy controls were obtained. The expression patterns of angiogenic factors and MMPs were analyzed by immunohistochemistry, protein chip array, and RT-qPCR.

**Results:**

Results showed higher expression of ANG, ANG-2, HGF, PIGF, VEGF, MMP-1, MMP-2, MMP-3, MMP-8, MMP-9, MMP-10, MMP-13, TIMP-1, and TIMP-2 in OSCC-tissues compared to the ANT. Among the overexpressed markers in OSCC-tissues, HGF, VEGF, PIGF, PDGF-BB, MMP-1, MMP-3, MMP-8, MMP-9, MMP-10, MMP-13, and TIMP-2 were significantly upregulated in the saliva of OSCC patients compared to healthy controls.

**Conclusions:**

The levels of HGF, VEGF, PIGF, MMP-1, MMP-3, MMP-8, MMP-9, MMP-10, MMP-13, and TIMP-2 were upregulated both in OSCC tissue and saliva of OSCC patients. Bioinformatic analysis revealed the correlation of these factors with patient survival and cancer functional states in head and neck cancer, indicating these factors as possible saliva-based non-invasive diagnostic/prognostic markers and therapeutic targets of OSCC.

**Supplementary Information:**

The online version contains supplementary material available at 10.1186/s12885-022-09630-0.

## Introduction

Oral squamous cell carcinoma (OSCC) is the most common oral cancer, accounting for 90% of all oral cancer [1]. Although many advances have been made in cancer treatment, the mortality rate of OSCC has remained unchanged. The 5-year survival rate is around 50% in intermediate patients and less than 20% in advanced patients [2, 3]. Surgery, radiotherapy, and chemotherapy are currently available therapy for OSCCs [4]. Surgical resection is considered to be a promising treatment strategy for early cancer [5]. At present, the diagnosis of OSCC mainly relies on clinical examination, imaging, and histological analysis of suspicious sites, but the lesion location of OSCC often occurs in hidden places [6], making it difficult to diagnose at an early stage.

Induced angiogenesis in OSCC had been reported to promote cancer progression and metastasis [7–10]. Vascular endothelial growth factor (VEGF), angiogenin (ANG), and platelet-derived growth factor (PDGF) signaling are reported as possible therapeutic targets in OSCC [11]. Similarly, angiogenic factors including hepatocyte growth factor (HGF) and placental growth factor (PIGF) had been reported to regulate OSCC invasion [12–14]. Furthermore, matrix metalloproteinases (MMPs) are a family of zinc ion and calcium ion-dependent proteins, which promote tumor progression and metastasis [15]. Therefore, it is essential to investigate the role of angiogenic factors and MMPs in OSCC progression.

Similar to blood, saliva is a complex body fluid known to contain several cellular and molecular components [16]. Saliva contacts directly with the oral mucosa and cancerous lesions thus are practicable for OSCC screening [17]. The altered level of certain molecules in saliva could be linked with oral and systemic diseases [18]. Saliva-based diagnosis is practical, non-invasive, and more accurate than available alternatives [19]. These advantages of saliva-based diagnosis may contribute to the early screening of many oral/systemic diseases and improve clinical management. It has been demonstrated that saliva is a useful diagnostic tool for assessing distant malignancies, including breast cancer [20], lung cancer [21], and Sjögren syndrome [22]. Similarly, salivary immunoglobulins have long been described as biomarkers for HIV infection [19]. Interleukin (IL)-1, IL-6, IL-8, VEGF, s90K/Mac-2 binding protein (M2BP), profilin, myeloid-related protein-14 (MRP14), and catalase have been reported as salivary biomarkers of oral cancer [18, 20–23]. Various angiogenic factors and MMPs are upregulated in OSCC tissue and serum. However, the expression pattern of angiogenic factors and MMPs in the saliva of OSCC patients is not fully understood.

In this study, we aimed to evaluate the expression pattern of various angiogenic factors and MMPs in OSCC tissue and saliva of OSCC patients to unravel the possible non-invasive diagnostic/prognostic markers and therapeutic targets of OSCC.

## Materials and Methods

### Patients and specimen collection

OSCC and adjacent normal tissues (ANT) of 10 OSCC patients were obtained. The study was approved by the medical ethics committee of Affiliated Stomatology Hospital of Guangzhou Medical University (Approval no. KY2019026). Informed consent was obtained from all subjects (patients and healthy controls). All the experimental protocols involving human samples were in accordance with the guidelines of the Declaration of Helsinki. Patients neither received radiotherapy nor chemotherapy before surgery. ANT sample was obtained from a 2 cm distance from the tumor tissue. Tissue specimens were stained with H&E staining to distinguish cancerous tissue from ANT. Saliva samples were collected from OSCC patients without chemotherapy or radiotherapy. Healthy controls without oral mucosal lesions, pulpitis, gingivitis, or periodontitis were recruited. OSCC patients and healthy controls had no other systemic and autoimmune diseases. Medication was stopped 3 days before the saliva collection. Similarly, smoking and alcohol consumption was prohibited 24 h before saliva collection. Subjects were adjusted to regulatory sleep and required not to eat or drink within 1–2 h after brushing their teeth in the morning. OSCC patients or healthy controls were instructed to spit unstimulated pooled saliva (around 4 ml) in a container. The collected saliva was centrifuged for 2 min at 10,000 rpm to acquire the supernatant. Saliva was collected from 8 OSCC patients and 8 healthy control and stored at -80˚C. And the tumor tissues were collected from 10 OSCC patients including 8 patients for saliva collection. Tissue and saliva were obtained from the same OSCC patients. The clinical characteristics and demographics of patients and healthy controls are shown in Table S1.

### Immunohistochemistry (IHC)

OSCC and normal tissues were fixed with 4% paraformaldehyde and embedded in paraffin, 5 µm thick tissue sections were deparaffinized and rehydrated in sequential xylene and gradient ethanol. The tissue sections were immersed in citrate buffer (pH 6.0) and heated for 30 min for antigen retrieval. After incubating with 3% H_2_O_2_, the tissue sections were blocked with bovine serum albumin (BSA, Serivicebio, China), followed by overnight incubation with the primary antibodies CD31 (Serivicebio, China, 1:300), CK18 (Abcam, USA, 1:200), MMP2 (Serivicebio, China, 1:1200), MMP3 (Serivicebio, China, 1:800), and MMP13 (Serivicebio, China, 1:200) at 4˚C. Tissue sections were then washed three times with PBS and incubated with goat anti-rabbit IgG (Serivicebio, China, 1:200) with HRP at room temperature. The bound antibody was then observed with diaminobezidin (DAB, Serivicebio, China), and hematoxylin. Substitution of the primary antibody with PBS was used as a negative control. CD31 staining was used to assess microvascular density (MVD) following the method described previously [24]. The micrograph of IHC was taken using PRECICE 500B ScanScope image analyzer (UNIC, China). The immunostained positive area was calculated in the epithelial basal cells, spinous cells, inflammatory cells, and fibroblasts as described previously [25]. IHC Profiler plugin in the ImageJ software was used for calculating the intensity of IHC. And the percentage of positive cells was calculated by ImageJ. The intensity scores were quantified as follows: 0 = negative, 1 = weak, 2 = moderate, 3 = strong. The proportion of immunopositive area was defined as follows: 0 = negative, 1 =  < 10%, 2 = 11–50%, 3 = 51–80%, and 4 =  ≥ 80% of positive cells. The final immunoreactive score was determined by multiplying the intensity and the proportion scores of the stained cells to obtain an immunoreactive score.

### Real-time quantitative PCR (RT-qPCR) analysis

Total RNA was extracted from OSCC tissue and ANT using TRIzol reagent (Invitrogen, USA) according to the manufacturer’s instructions. The cDNA was generated in the T100 gradient PCR machine (Bio-rad, USA) using a PrimeScript RT reagent kit with gDNA Eraser (Takara, Japan). RT-qPCR was performed using TB Green Premix Ex Taq II Kit (Takara, Japan) on an AriaMx Real-time quantitative PCR machine (Agilent, USA). The PCR reaction conditions were 95℃ for 30 s, followed by 40 cycles at 95℃ for 5 s and 60℃ for 30 s. Each reaction was performed in triplicate. The expressions of the angiogenic genes including ANG, bFGF, EGF, HGF, HB-EGF, VEGF, PDGF-BB, Leptin, and MMPs including MMP-1, MMP-2, MMP-3, MMP-8, MMP-9, MMP-10, MMP-13, TIMP-1, TIMP-2, and TIMP-4 were analyzed by RT-qPCR. GAPDH gene was used as the internal control. The fold change relative to the control group was measured by the 2^−∆∆Ct^ method. Primer sequences used in this study are listed in Table S2.

### Protein chip array

Tissue and saliva samples from patients were used to measure the expression of ANG, angiopoietin-2 (ANG-2), bFGF, heparin-binding EGF (HB-EGF), HGF, LEP, PDGF-BB, PIGF, and VEGF by the Quantibody® Human Angiogenesis Array 1 (RayBiotech, USA) and MMP-1, MMP-2, MMP-3, MMP-8, MMP-9, MMP-10, MMP-13, TIMP-1, and TIMP-2 by the Quantibody® Human MMP Array 1 (RayBiotech, USA). The detection was performed according to the manufacturer's instructions. Briefly, supernatant from centrifuged tissue samples treated with cell lysate and centrifuged saliva samples were used. The glass slide was completely air dry and blocked by a blocking solution for 1 h. The tissue sample diluent 100 µL (500 µg/mL) and 80 µL of the diluted tissue lysate or saliva samples were separately added to the slides and incubated overnight at 4˚C. The samples diluted were decanted from the wells and washed with wash buffer at room temperature with gentle rocking. The detection antibody cocktail was added to each well and incubated at room temperature, followed by a washing step. Afterward, Cy3 equivalent dye-conjugated streptavidin was added and incubated in a dark room, followed by a washing step. Fluorescence signals were obtained by an InnoScan 300 Microarray Scanner (Innopsys, France) at 532 nm wavelength and 10 µm resolution. Data were analyzed using Mapix (Innopsys, France) analysis software.

### Survival analysis

The expression data of head and neck squamous carcinoma for angiogenic-related and MMPS was downloaded from the cancer genome atlas database (TCGA). The patients’ information is shown in Table S3. The survival curve for 260 HNSCC patients with higher expression of angiogenic factors/MMPs and other 259 patients with lower angiogenic factors/MMPs expression was plotted to evaluate the 5-Year survival percentage and duration.

### Correlation analysis of angiogenic factors and MMPs expression with the functional states in head and neck squamous cell carcinoma (HNSCC)

Average correlations data between genes of interest and functional states in HNSCC patients were downloaded from CancerSEA (http://biocc.hrbmu.edu.cn/CancerSEA). CancerSEA is a dedicated database that aims to comprehensively decode distinct functional states of cancer cells at the single-cell level. The expression pattern of ANGs and MMPs and their correlation with 14 crucial biological functions in HNSCC, including cancer cell stemness, invasion, metastasis, proliferation, EMT, angiogenesis, apoptosis, cell cycle, cell differentiation, DNA damage, DNA repair, hypoxia, inflammation, and quiescence were analyzed.

### Statistical analysis

The results were represented as the mean ± standard deviation (SD). The statistical significance was determined by Student’s t-test at p < 0.05. Cox proportional hazards models were used for the survival analysis by considering ages, sex, tumor sites, alcohol/smoking status, and tumor stages. The graphs were plotted using GraphPad Prism 7.0.

## Results

### Histology and immunohistochemistry of angiogenic factors in ANT and OSCC tissues

**Fig. 1:**
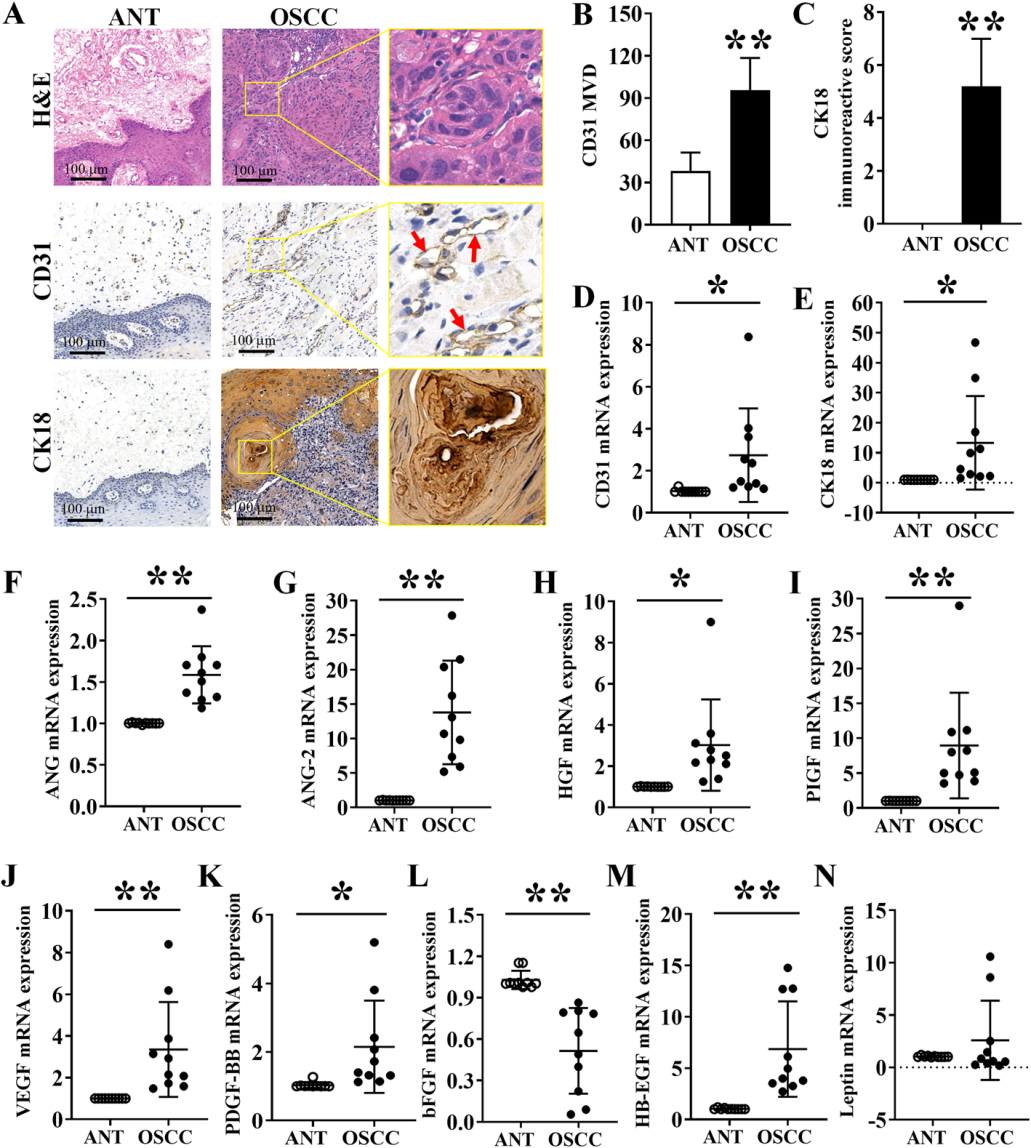
Histology and IHC analysis of angiogenic markers in OSCC tissues and ANT. (**A**) H&E-stained histological as well as CD31 and CK18 IHC images of human OSCC tissue and ANT sections. (**B**) Quantitative analysis of MVD in the CD31-stained area. (**C**) CK18 positive area in ANT and OSCC tissue (*n* = 5). Relative mRNA expression of (**D**) CD31, (**E**) CK18, (**F**) angiogenin, (**G**) ANG-2, (**H**) HGF, (**I**) PIGF, (**J**), VEGF, (**K**) PDGF-BB, (**L**) bFGF, (**M**) HB-EGF, and (**N**) leptin in ANT and OSCC tissue. Data are presented as mean ± SD, *n* = 10. Significant differences compared to the ANT group, *p < 0.05 and **p < 0.01

H&E stained ANT histological tissue sections showed normal oral tissue histology. In contrast, H&E stained OSCC tissue sections showed a distinct pattern of well-differentiated OSCC (Fig. 1A). Tissue sections showed few CD31 stained areas in ANT sections, and distinct CD31-stained microvessels in OSCC tissue sections (Fig. 1A). CK18 is a soluble cytokeratin present in proliferating cells with a high expression in the G2-M phase of the cell cycle [26]. Since CK18 is highly expressed in OSCC tissues, we further performed CK18 immunohistochemistry. Brown granular staining in the nucleus of tumor cells was considered positive for CK18. ANT sections were hardly stained with CK18, but OSCC tissue sections showed intense CK18 staining (Fig. 1A). Quantitative analysis of CD31 stained microvessel structure revealed 2.5-fold higher MVD in OSCC tissue compared to ANT (Fig. 1B). Similarly, OSCC tissue showed a 5.06-fold higher expression of CK18 compared to ANT (Fig. 1C). mRNA expression analysis by RT-qPCR also showed upregulation of CD31 and CK18 in OSCC tissue compared to ANT (Fig. 1D and E).

### mRNA expression of angiogenic factors in OSCC tissues and ANT

We further analyzed the mRNA level expression of angiogenic factors in OSCC and ANT tissue by RT-qPCR. The expressions of ANG, ANG-2, HGF, PIGF, VEGF, PDGF-BB, and HB-EGF in the OSCC-group were 1.58-, 13.56-, 3.00-, 8.86-, 3.34-, 2.08-, and 4.63-fold higher, respectively compared to ANT-group (Fig. 1F-Fig. 1K, Fig. 1M). LEP expression in OSCC-group was not significantly affected compared to the ANT group (Fig. 1N). The expression of bFGF was reduced in OSCC-group compared to the ANT group (Fig. 1L).

### Protein level expression of angiogenic factors in OSCC tissues and ANT

**Fig. 2:**
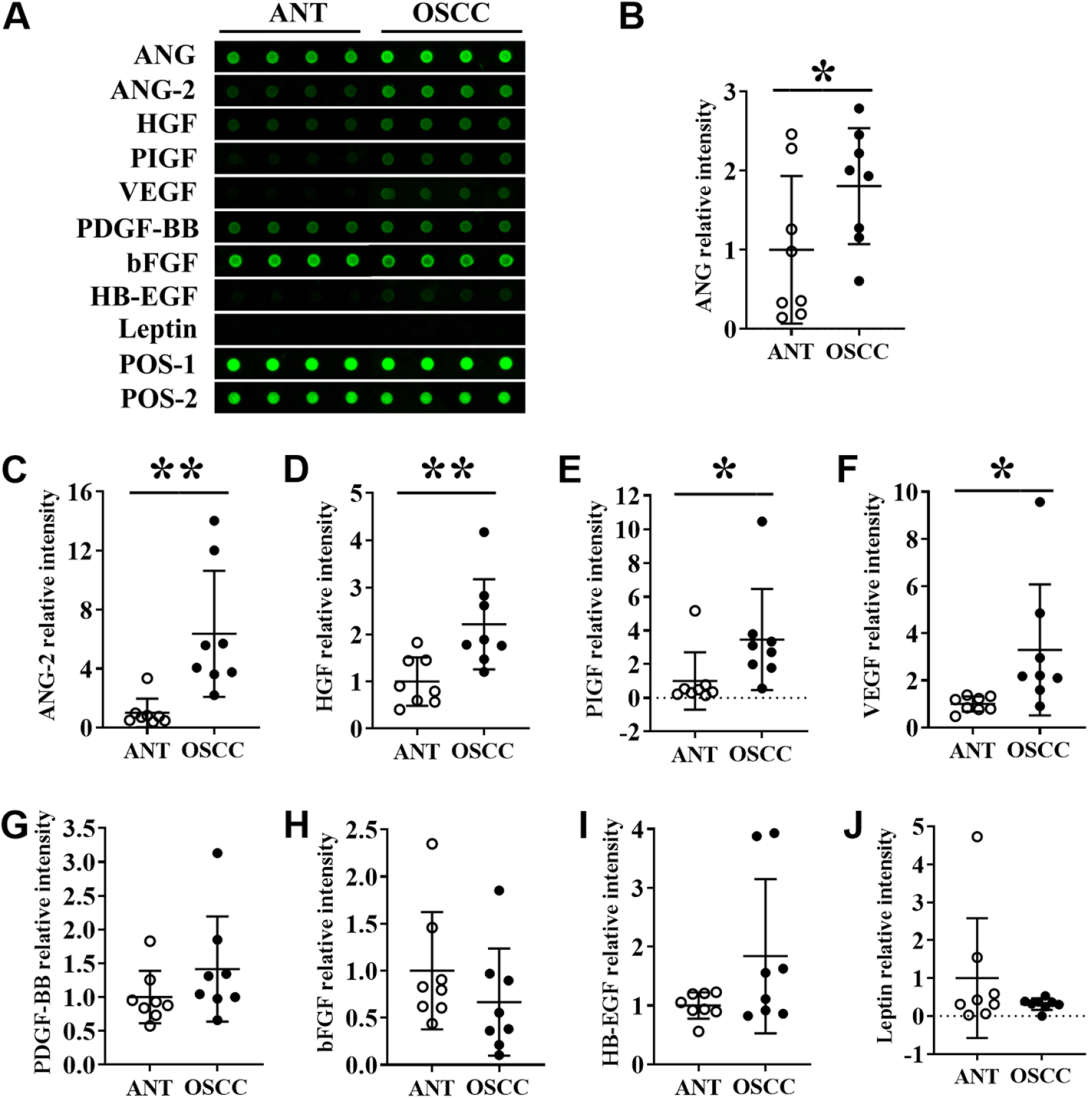
Angiogenic markers’ protein level expression analysis in OSCC tissues and ANT by protein array. (**A**) Representative protein array images showing the expression pattern of angiogenesis factors in ANT and OSCC tissue lysates. Quantitative analysis of (**B**) angiogenin, (**C**) ANG-2, (**D**) HGF, (**E**) PIGF, (**F**) VEGF, (**G**) PDGF-BB, (**H**) bFGF, (**I**) HB-EGF, and (**J**) leptin expression from protein array images. Data are presented as mean ± SD, *n* = 8. Significant differences compared with the ANT group, *p < 0.05 and **p < 0.01

We analyzed the protein level expression of angiogenic factors in OSCC and ANT tissue lysates by protein chip array. Among the angiogenic factors tested, the fluorescence intensities of angiogenin, ANG, ANG-2, HGF, PIGF, and VEGF were prominent in OSCC-group compared to the ANT group (Fig. 2A). Quantitative analysis revealed 1.80-, 6.37-, 2.21-, 3.47-, and 3.30-fold higher expression of ANG, ANG-2, HGF, PIGF, and VEGF in the OSCC-group, respectively compared to ANT-group (Fig. 2B-Fig. 2F). Expressions of PDGF-BB, bFGF, HB-EGF, and leptin were not significantly changed in OSCC tissue compared to ANT (Fig. 2G-Fig. 2J).

### Protein level expression of angiogenic factors in the saliva of OSCC patients and healthy controls

**Fig. 3:**
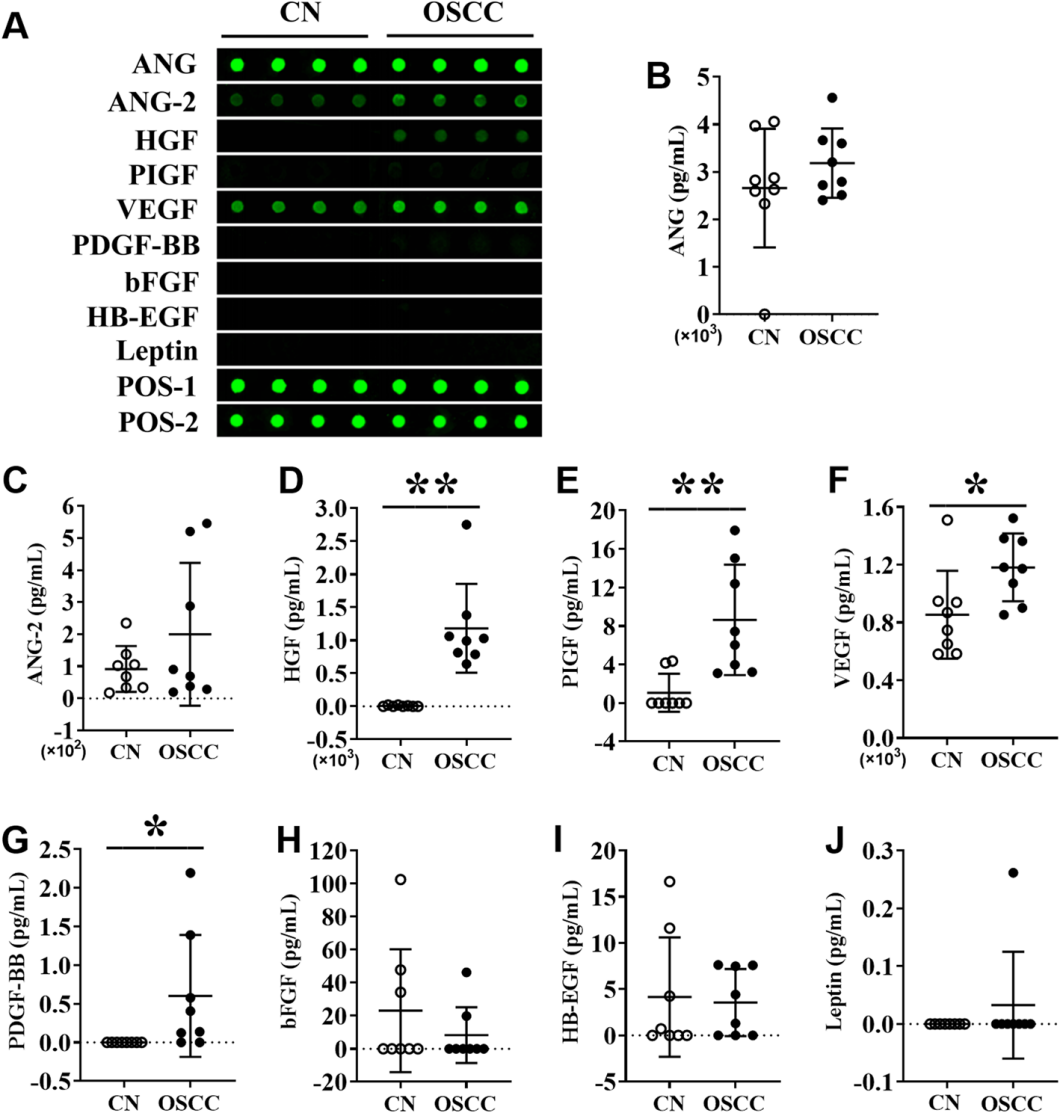
Angiogenic markers’ protein level expression analysis in the saliva of OSCC patients and healthy controls. (**A**) Representative protein array images showing the expression pattern of angiogenesis factors in the saliva of healthy controls and OSCC patients. Quantitative analysis of (**B**) angiogenin, (**C**) ANG-2, (**D**) HGF, (**E**) PIGF, (**F**) VEGF, (**G**) PDGF-BB, (**H**) bFGF, (**I**) HB-EGF, and (**J**) leptin expression from protein array images. Data are presented as mean ± SD, *n* = 8. Significant differences compared with the healthy control group, *p < 0.05 and **p < 0.01

We further analyzed the protein level expression of angiogenic factors in the saliva of OSCC patients and healthy controls. The main aim of this experiment was to further determine the potential application of angiogenic factors as non-invasive diagnostic and prognostic markers in OSCC. Among the angiogenic markers tested, the expressions of HGF, PIGF, and VEGF were 2.00-, 8.10-, and 1.38-fold higher in the saliva of OSCC patients compared to healthy controls (Fig. 3D-Fig. 3F). Interestingly, PDGF-BB was not detected in healthy controls, while 62.5% of OSCC patients showed higher expression of PDGF-BB in saliva (Fig. 3G). Expressions of ANG, ANG-2, bFGF, HB-EGF, and leptin in saliva were not significantly different in OSCC compared to control (Fig. 3A-Fig. 3C, Fig. 3H-Fig. 3J). Expressions of PIGF, PDGF-BB, bFGF, HB-EGF, and leptin in saliva were relatively low to visualize the fluorescence intensity by the naked eye. However, fluorescence signals of these proteins were obtained by an InnoScan 300 Microarray Scanner and quantified.

### Immunohistochemistry of MMPs in ANT and OSCC tissues

**Fig. 4:**
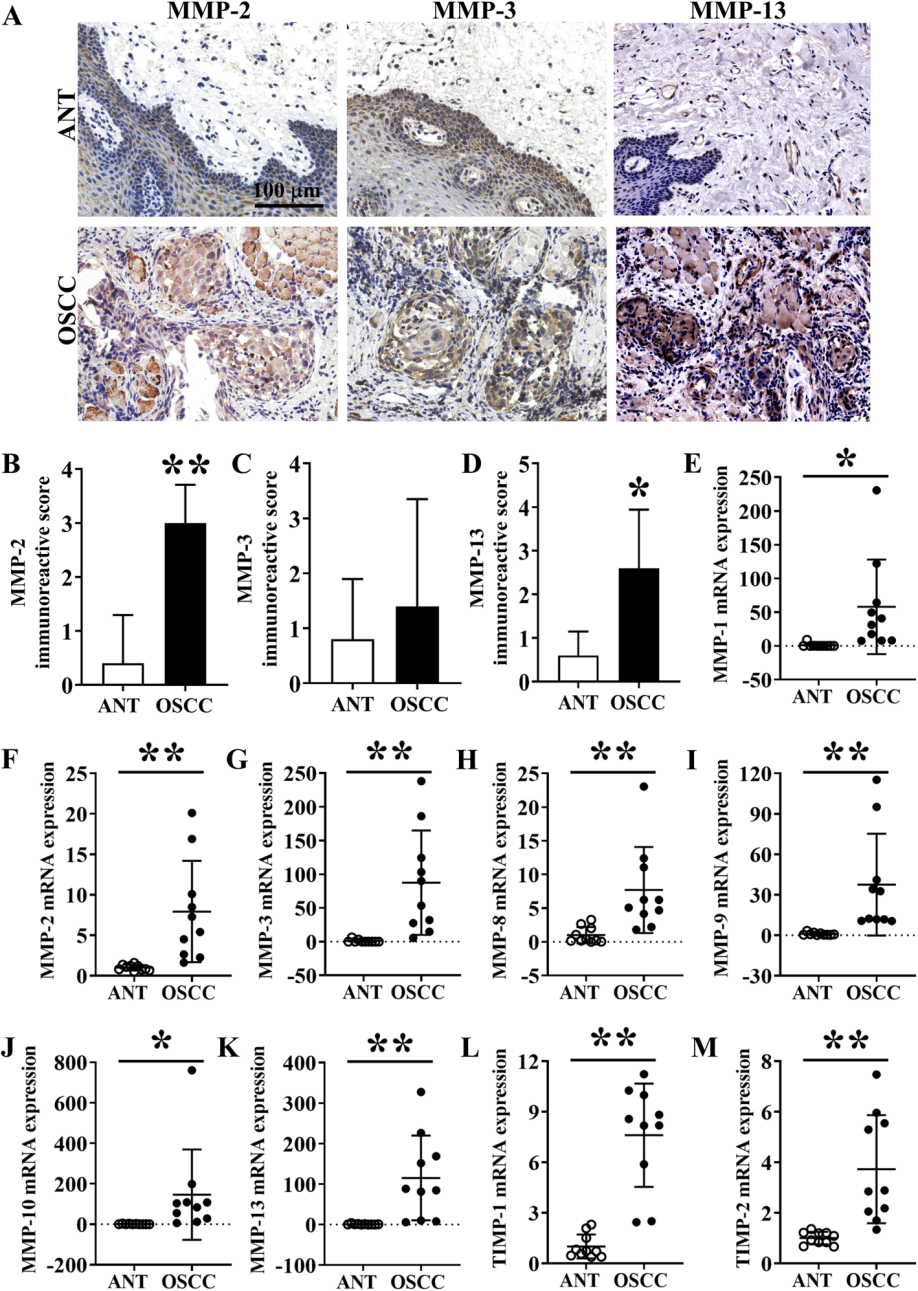
MMPs expression analysis by IHC in OSCC tissues and ANT. (**A**) MMP-2, MMP-3, and MMP-13 IHC images of ANT and OSCC tissue sections. Quantification of (**B**) MMP-2, (**C**) MMP-3, and (**D**) MMP-13 positive area in ANT and OSCC tissue sections, *n* = 5. Relative mRNA expression of (**E**) MMP-1, (**F**) MMP-2, (**G**) MMP-3, (**H**) MMP-8, (**I**) MMP-9, (**J**) MMP-10, (**K**) MMP-13, (**L**) TIMP-1, and (**M**) TIMP-2 in ANT and OSCC tissue, *n* = 10. Data are presented as mean ± SD. Significant differences compared with the ANT group, *p < 0.05 and **p < 0.01

In addition, the expression pattern of MMPs in ANT and OSCC tissues was evaluated. IHC images showed more clear and distinct staining of MMP-2 and MMP-13 in OSCC tissue sections compared to ANT sections (Fig. 4A). There was not much difference in MMP-3 staining in ANT and OSCC groups. The negative control of IHC staining of MMP13 for ANT and OSCC was shown in Fig. S1. According to quantitative analysis data, MMP-2 and -13 showed significant differences between ANT and OSCC, in contrast to MMP-3 which did not reach statistical significance (*P* = 0.86) (Fig. 4B-Fig. 4D).

### mRNA expression of MMPs in OSCC tissues and ANT

We further analyzed the mRNA level expression of MMPs in OSCC and ANT tissue by RT-qPCR. The expressions of MMP-1, MMP-2, MMP-3, MMP-8, MMP-9, MMP-10, MMP-13, TIMP-1, and TIMP-2 in OSCC-group were 58.01-, 7.92-, 87.54-, 7.69-, 37.51-, 146.29-, 115.20-, 7.60-, and 3.73-fold higher, respectively compared to ANT-group (Fig. 4E-Fig. 4M).

### Protein level expression of MMPs in OSCC tissues and ANT

**Fig. 5:**
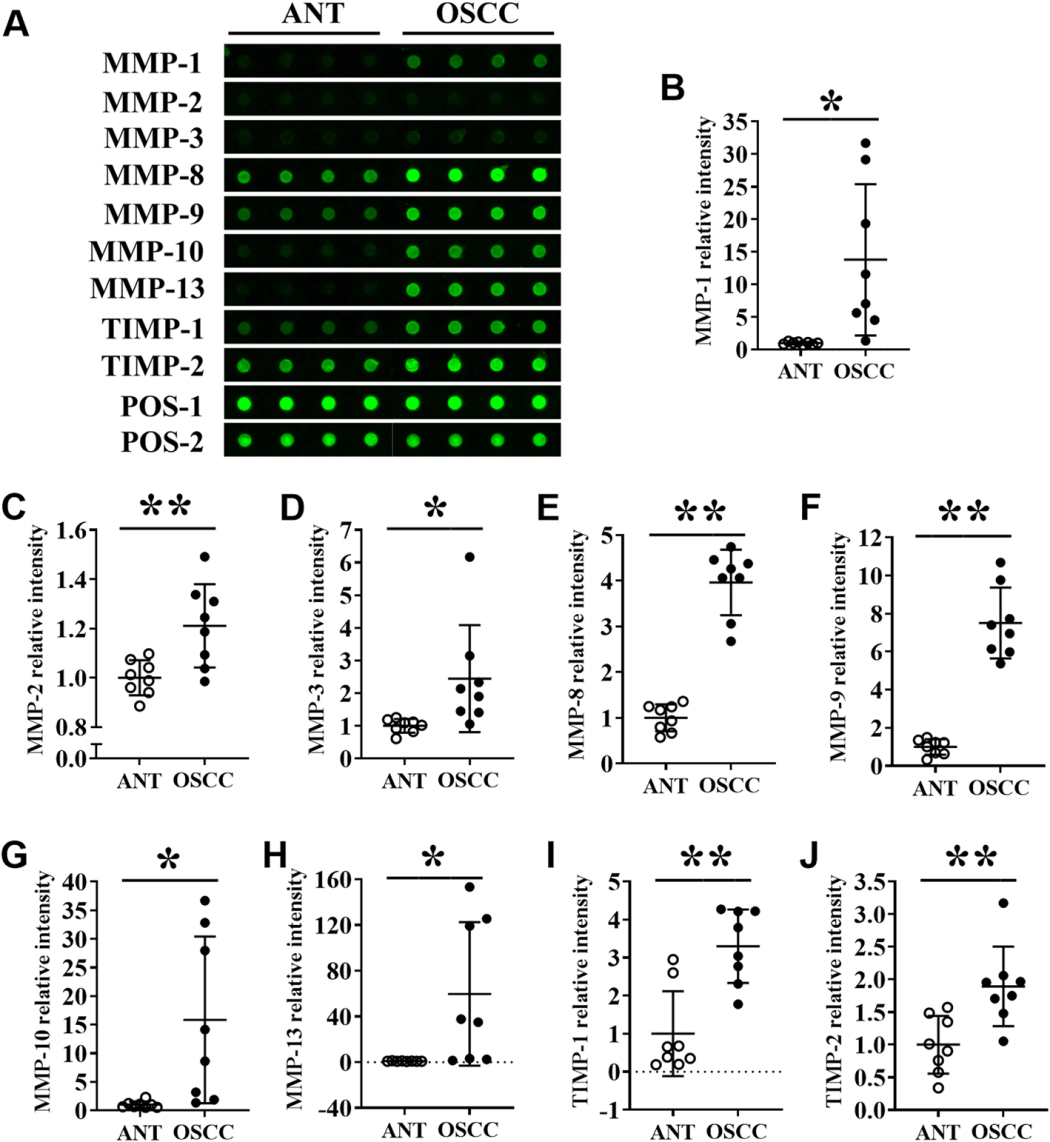
MMPs protein level expression analysis in OSCC tissues and ANT by protein array. (**A**) Representative protein array images showing the expression pattern of MMPs in ANT and OSCC tissue lysates. Quantitative analysis of (**B**) MMP-1, (**C**) MMP-2, (**D**) MMP-3, (**E**) MMP-8, (**F**) MMP-9, (**G**) MMP-10, (**H**) MMP-13, (**I**) TIMP-1, and (**J**) TIMP-2 expression from protein array images. Data are presented as mean ± SD, *n* = 8. Significant differences compared with the ANT group, *p < 0.05 and **p < 0.01

To determine the protein levels of MMPs in tissues, we performed a semi-quantitative protein chip array. The results showed that the fluorescence intensities of MMP-1, MMP-2, MMP-3, MMP-8, MMP-9, MMP-10, MMP-13, TIMP-1, and TIMP-2 in the OSCC group were significantly increased compared with the ANT-group (Fig. 5A). The mean fluorescence intensities of MMP-1, MMP-2, MMP3, MMP-8, MMP-9, MMP-10, MMP-13, TIMP-1 and TIMP-2 in OSCC tissue were 13.80-, 1.21-, 2.44-, 3.96-, 7.50-, 15.83-, 59.75-, 3.29-, and 1.89-fold higher than ANT-group, respectively (Fig. 5B-Fig. 5J). The result of protein level expression of MMPs was in accordance with the results of RT-qPCR (Fig. 4).

### Protein level expression of MMPs in the saliva of OSCC patients and healthy controls

**Fig. 6:**
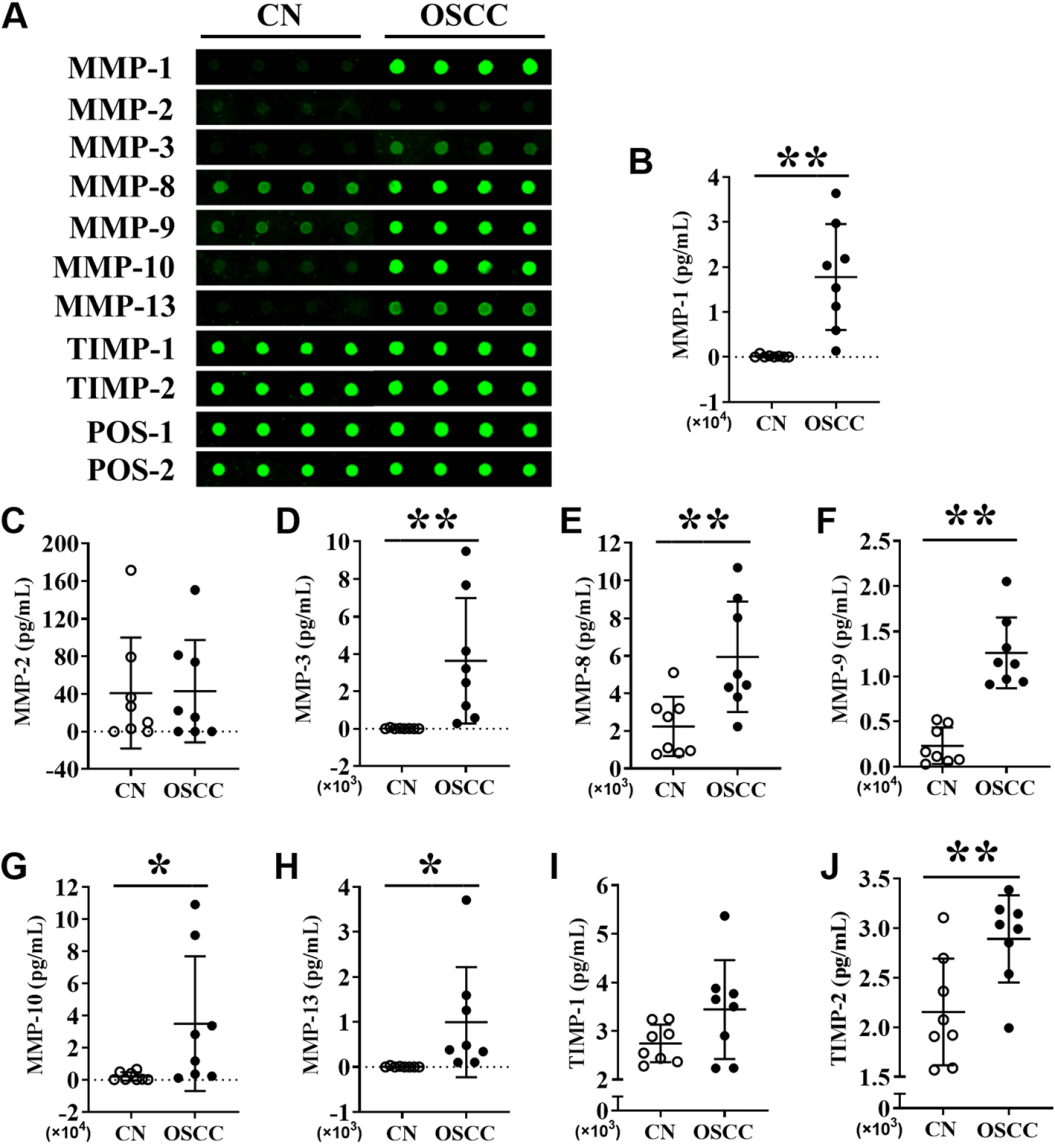
MMPs protein level expression analysis in the saliva of OSCC patients and healthy controls. (**A**) Representative protein array images showing the expression pattern of MMPs in the saliva of healthy controls and OSCC patients. Quantitative analysis of (**B**) MMP-1, (**C**) MMP-2, (**D**) MMP-3, (**E**) MMP-8, (**F**) MMP-9, (**G**) MMP-10, (**H**) MMP-13, (**I**) TIMP-1, and (**J**) TIMP-2 expression from protein array images. Data are presented as mean ± SD, *n* = 8. Significant differences compared with the healthy control group, *p < 0.05 and **p < 0.01

The protein level expression of MMPs in the saliva of OSCC patients and healthy controls was further analyzed. It demonstrated that the expression levels of MMP-1, MMP-3, MMP-8, MMP-9, MMP-10, and MMP-13 in saliva were relatively high to visualize the green fluorescence (Fig. 6A). Quantification analysis revealed that the levels of MMP-1, MMP-3, MMP-8, MMP-9, MMP-10, MMP-13, and TIMP-2 were upregulated by 111.71-, 256.32-, 2.66-, 5.48-, 16.31-, 174.20-, and 1.34-fold in the saliva of OSCC patients, respectively, compared to healthy controls (Fig. 6B, Fig. 6D-Fig. 6H and Fig. 6J). Expressions of MMP2 and TIMP-1 in saliva were not significantly different in OSCC compared to control (Fig. 6C, Fig. 6I). Intriguingly, MMP-1, MMP-3, and MMP-13 were highly expressed in the saliva of the OSCC group, but their expressions were too low to be detected in healthy individuals.

### The correlation between angiogenic factors and MMPs expression with the functional states in HNSCC

**Fig. 7:**
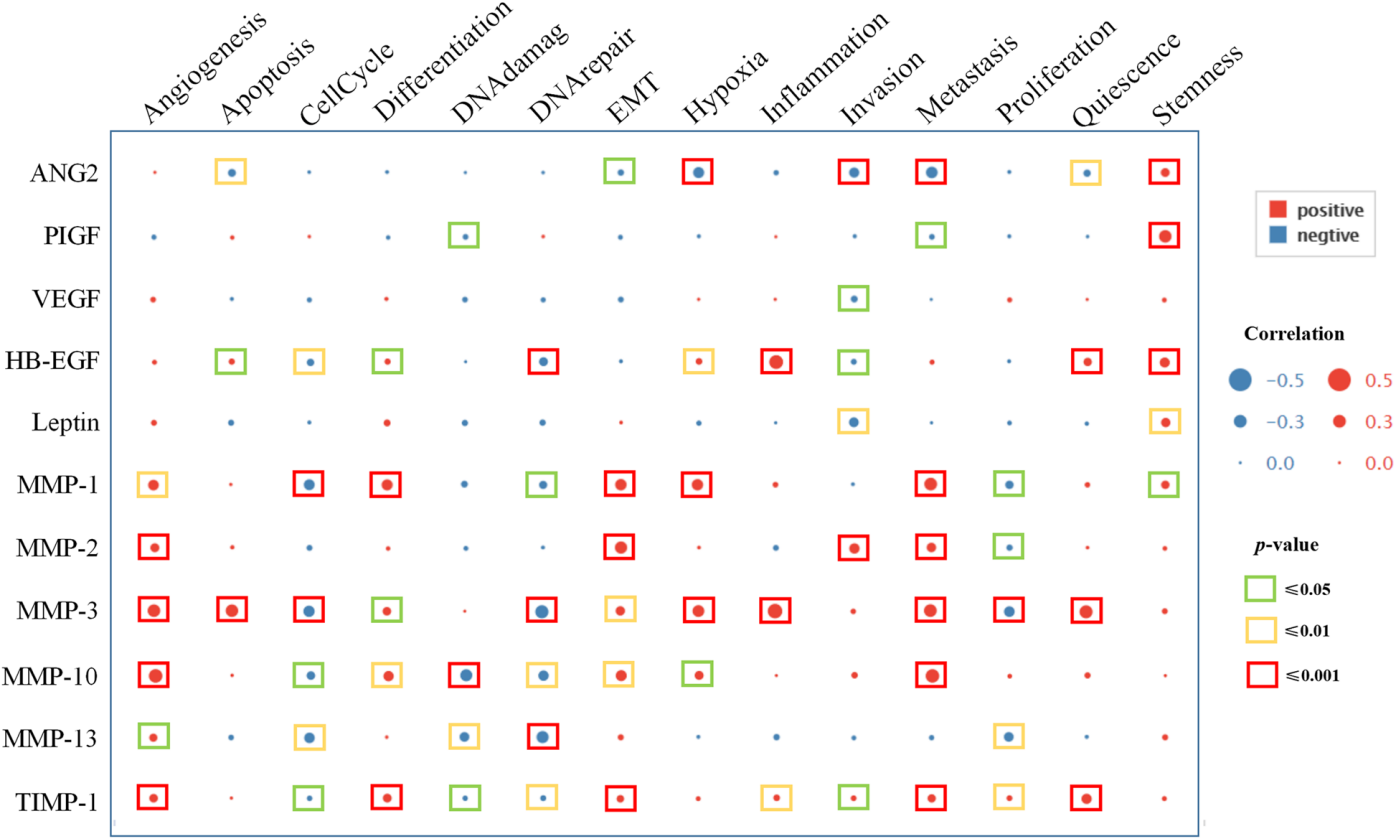
Correlation analysis of ANGs and MMPs expressions with HNSCC tumor cell functions by CancerSEA. Functional states mainly include angiogenesis, apoptosis, cell cycle, differentiation, DNA damage, DNA repair, EMT, hypoxia, inflammation, invasion, metastasis, proliferation, quiescence, and stemness. EMT: endothelial mesenchymal transition

Figure 7 depicts the correlation between the expression patterns of ANGs and MMPs expression with the functional states in HNSCC at the single-cell level. ANG2 and PIGF expression positively correlated with cancer cell stemness. HB-EGF expression positively correlated with inflammation, cancer cell quiescence, and stemness. The expression of leptin positively correlated with cancer cell stemness. MMPs expression was positively correlated with angiogenesis, EMT, and metastasis. Intriguingly, HB-EGF, MMP-1, MMP-3, MMP-10, MMP-13, and TIMP-1 showed a negative correlation with cell cycle and DNA repair. While, the expressions of MMP-10, MMP-13, and TIMP-1 were negatively correlated with DNA damage. And the higher expression of ANG-2 was negatively correlated with invasion, metastasis, and hypoxia.

### Survival analysis

**Fig. 8:**
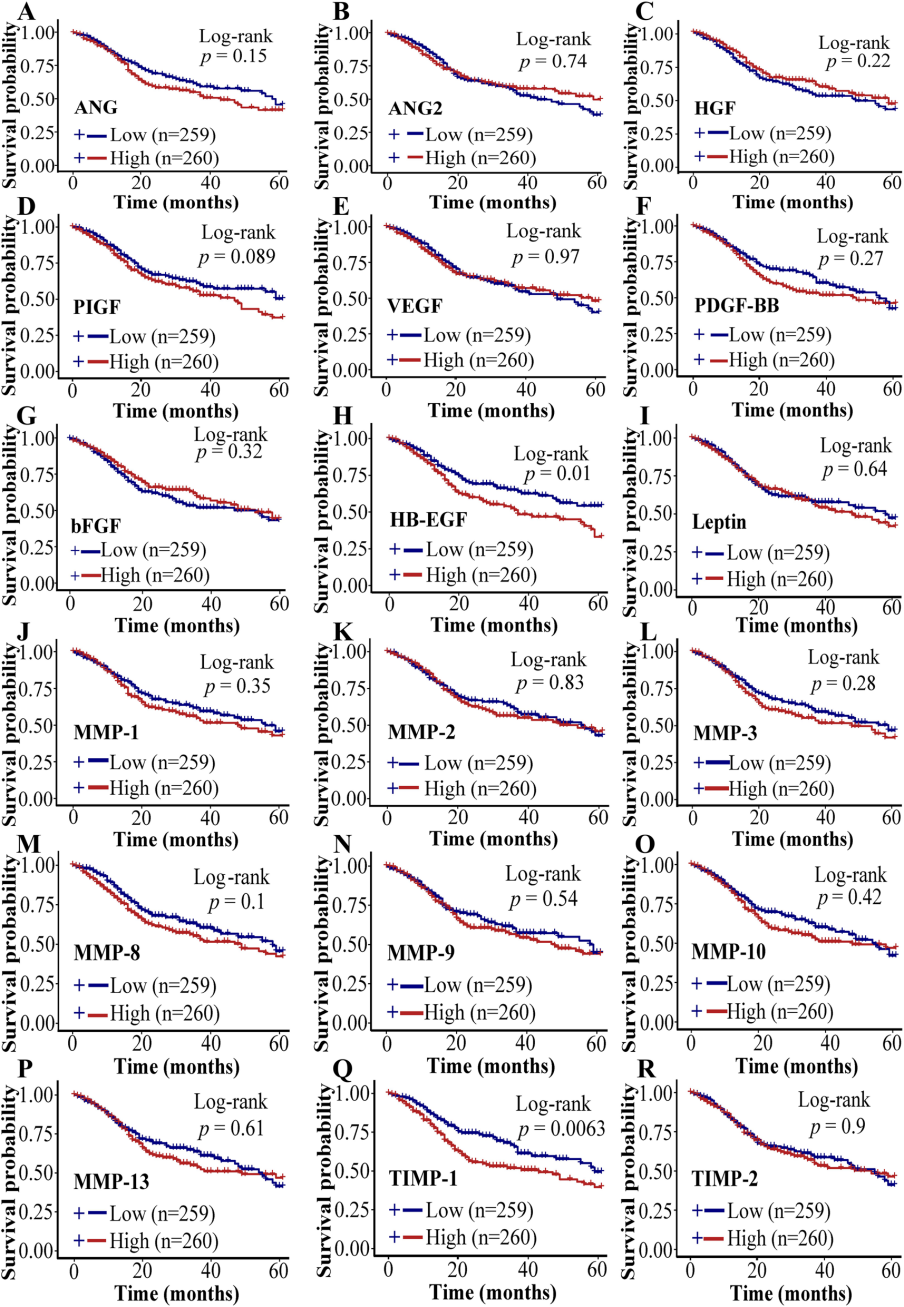
Correlation analysis of ANGs and MMPs expressions with HNSCC patient survival. Correlation of (**A**) ANG, (**B**) ANG-2, (**C**) HGF, (**D**) PIGF, (**E**) VEGF, (**F**) PDGF-BB, (**G**) bFGF, (**H**) HB-EGF, (**I**) Leptin, (**J**) MMP-1, (**K**) MMP-2, (**L**) MMP-3, (**M**) MMP-8, (**N**) MMP-9, (**O**) MMP-10, (**P**) MMP-13, (**Q**) TIMP-1, or (**R**) TIMP-2 expression pattern with HNSC patient survival rate and duration (248 OSCC patients). *p < 0.05 and **p < 0.01

We further analyzed the survival curve from 260 HNSCC patients (50%) with higher expressions of ANGs and MMPs and 249 patients (50%) with lower expressions of ANGs and MMPs. HNSCC patients with lower expressions of HB-EGF (*p* = 0.01), TIMP-1 (*p* = 0.0063) showed a significantly higher 5 years survival rate compared to those HNSCC patients with higher expressions of these factors (Fig. 8 D, Fig. 8Q). Similarly, HNSCC patients with lower expressions of ANG (*p* = 0.15), Leptin (*p* = 0.64), PIGF (*p* = 0.089), MMP- MMP-1 (*p* = 0.35), MMP-3 (*p* = 0.28), MMP-8 (*p* = 0.1), and MMP-9 (*p* = 0.54) showed higher 5 years survival rate compared to those HNSCC patients with higher expressions of these factors but there was no significant difference between lower and higher expression groups (Fig. 8A, Fig. 8F, Fig. 8H, Fig. 8J, Fig. 8L-Fig. 8N). HNSCC patients with higher expression of ANG-2 (*p* = 0.74), bFGF (*p* = 0.32), HGF (*p* = 0.22), VEGF (*p* = 0.97), PDGF-BB (*p* = 0.27), MMP-2 (*p* = 0.83), MMP-10 (p = 0.42), MMP-13 (*p* = 0.61), and TIMP-2 (*p* = 0.9) showed higher 5 years survival rate compared to those HNSCC patients with higher expressions of these factors but there was no significant difference between lower and higher expression groups (Fig. 8B, Fig. 8C, Fig. 8E, Fig. 8G, Fig. 8I, Fig. 8K, Fig. 8O, Fig. 8P, Fig. 8R).

## Discussion

**Fig. 9:**
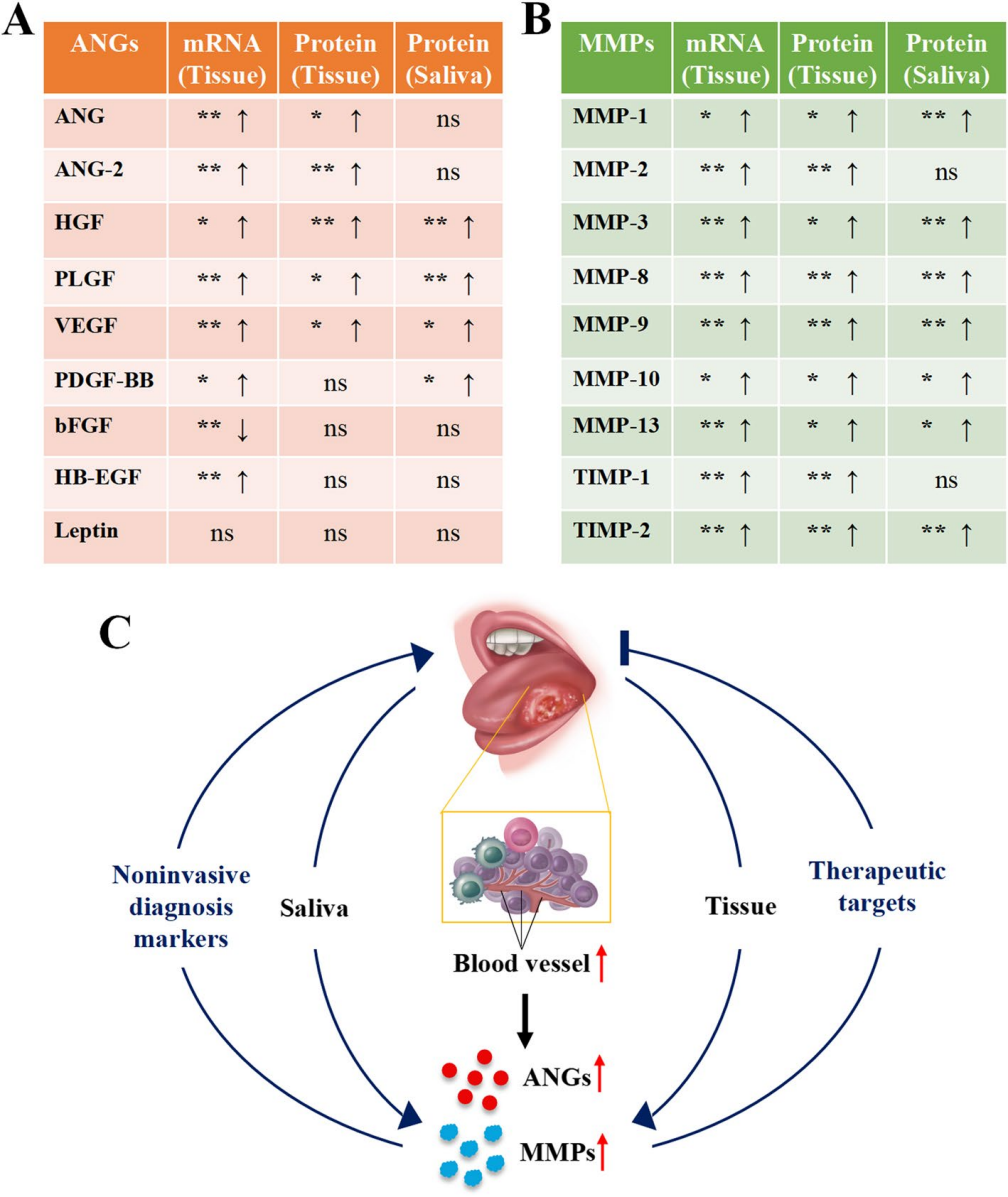
Scheme showing ANGs and MMPs expression based on protein chip array and their possible application in OSCC diagnosis and treatment. (**A**) ANGs and MMPs in the OSCC may serve as non-invasive diagnostic markers and therapeutic targets. (**B**) The expression of ANGs in tissues and saliva. (**C**) The expression of MMPs in tissues and saliva. *p < 0.05 and **p < 0.01

Identification of novel biomarkers for OSCC diagnosis/prognosis helps early diagnosis and treatment and decreases patient morbidity and mortality. Considering the importance of angiogenesis and MMPs in OSCC development and metastasis, it is wise to explore the expression patterns of angiogenic factors and MMPs. Results of this study found a direct correlation between angiogenesis with OSCC development. This study found higher expression of HGF, VEGF, PIGF, PDGF-BB, MMP-1, MMP-3, MMP-8, MMP-9, MMP-10, MMP-13, and TIMP-2 both in OSCC tissue and saliva of OSCC patients based on the protein chip-array. IHC and protein chip array of some of the factors did not give the same results. Our findings indicate these factors have the potential as saliva-based non-invasive possible diagnostic/prognostic markers and therapeutic targets of OSCC (Fig. 9).

Histological analysis is the gold standard for OSCC diagnosis. No tumor progression in ANT-tissue was verified by histological analysis. CD31, an endothelial marker, is highly expressed in various cancer tissues, including OSCC [27, 28]. In this study, CD31 is highly expressed in the connective tissue of OSCC samples compared to ANT. The higher degree of angiogenesis promotes not only OSCC progression but also cancer invasion [29]. CK18, a cytokeratin protein, is highly expressed in proliferating cancer cells, including OSCC [30]. We found higher MVD in OSCC tissue compared to ANT. Similarly, the expression pattern of CK18 was in accordance with the higher CD31 expression and angiogenesis in OSCC. These findings indicate the key role of angiogenesis in OSCC progression.

ANG, ANG-2, HGF, PIGF, PDGF-BB, HB-EGF, VEGF, bFGF, and leptin are key growth factors that regulate angiogenesis in pathophysiological conditions [31]. In this study, we evaluated the expression of all these angiogenic growth factors in OSCC tissue and saliva of OSCC patients. The mRNA levels of ANG, ANG-2, HGF, PIGF, PDGF-BB, HB-EGF, and VEGF were upregulated in OSCC tissues compared to paired-ANT. Furthermore, the protein levels of ANG, ANG-2, HGF, PIGF, and VEGF were significantly increased in OSCC tissues. Saliva possesses tremendous potential in disease diagnosis due to its critical advantages, including minimum cost, non-invasiveness, and easy collection [32]. HGF, PIGF, and VEGF had been reported as therapeutic targets in OSCC [12–14, 33]. HGF regulates gastric cancer progression and metastasis [34]. In various cancers, PIGF is involved in tumor immune escape and metastasis [35]. Polz-Dacewicz and colleagues had reported the higher expression of VEGF in serum and saliva of oropharyngeal squamous cell carcinoma patients [36]. PDGF-BB is overexpressed in OSCC tissues and correlates with tumorigenesis and poor prognosis of OSCC [37]. In this study, HGF, PIGF, VEGF, and PDGF-BB were found to be significantly upregulated in OSCC tissue as well as in the saliva of the patients. Future research on the role of HGF, PIGF, VEGF, and PDGF-BB in OSCC progression, and metastasis could be useful to unravel these factors as novel diagnostic and therapeutic markers. This is the first study to show the higher expression of HGF, PIGF, and PDGF-BB in the saliva of OSCC patients. Our findings indicate that the angiogenic markers HGF, PIGF, VEGF, and PDGF-BB could be saliva-based novel diagnostic markers of OSCC.

MMPs participate in tumor progression, and metastasis, thereby hold diagnostic and therapeutic applications potential [38, 39]. MMP-1/protease-activated receptor-1 (PAR1) signaling axis plays an essential role in tumor angiogenesis by inducing vascular permeability [40]. Serum and salivary MMP1, MMP2, MMP-9, MMP-10, MMP-12, and MMP13 had been reported to upregulate in OSCC [41–44]. Moreover, MMP-9 triggers the angiogenic switch by releasing VEGF during tumorigenesis [45]. We found that the mRNA and protein levels of MMP-1, MMP-2, MMP-3, MMP-8, MMP-9, MMP-10, MMP-13, TIMP-1, and TIMP-2 were significantly increased in OSCC tissue compared to paired-ANT. Furthermore, the levels of MMP-1, MMP-3, MMP-8, MMP-9, MMP-10, MMP-13, and TIMP-2 were significantly upregulated in the saliva of OSCC patients compared to the healthy ones. Accordingly, the expressions of MMP-1, MMP-3, MMP-8, MMP-9, MMP-10, MMP-13, and TIMP-2 were upregulated both in OSCC tissue and in the saliva of OSCC patients. MMP-1, MMP-2, MMP-3, MMP-9, MMP-13, TIMP-1, and TIMP-2 had been reported to overexpress in HNSCC tissue compared with normal tissue. Thus, our data is consistent with previous reports [25, 46]. This is the first study to report the overexpression of salivary MMP-3, MMP-8, and TIMP-2 in OSCC suggesting these markers as saliva-based potential novel diagnostic/prognostic markers as well as therapeutic targets of OSCC. The HGF and MMP-9 content in saliva are quite different between healthy control and OSCC patients. In the present study, the highest HGF concentration in the control group saliva was 20.079 pg/mL, while the lowest in the OSCC group was 641.457 pg/mL. Similarly, the highest concentration for MMP9 in the control group was 5224.613 pg/mL, while the lowest in the OSCC group was 9135.693 pg/mL. More normal and tumor saliva samples should be collected and set the positive or negative cut-off value of HGF and MMP9.

In this study, we re-confirmed higher angiogenesis in human OSCC tissue. We analyzed the expression pattern of a panel of angiogenic factors and MMPs in OSCC tissue and saliva of OSCC patients. RT-qPCR, immunohistochemistry, and protein chip array analysis were used to extensively analyze the expression of the aforementioned markers in mRNA and protein levels in OSCC tissue and saliva. This study revealed the overexpression of HGF, PIGF, PDGF-BB, MMP-3, MMP-8, and TIMP-2 both in serum and saliva of OSCC patients for the first time. Moreover, the survival analysis of HNSCC showed a direct correlation between the higher expression of the HB-EGF and TIMP-1 expression with the lower survival rate of the patients. Moreover, the expression patterns of TIMP-1 in HNSCC positively correlated with angiogenesis, EMT, and metastasis. While HB-EGF was positively related to cancer inflammation and stemness. Although these are the results from HNSCC patients, the similarity between HNSCC and OSCC provides a hint about the functions of ANGs and MMPs in OSCC. The survival analysis and cancer cell functional states correlation with ANGs/MMPs expression are needed to confirm the exact roles of ANGs and MMPs in OSCC development and progression.

The overexpression of VEGF, MMP-1, MMP-2, MMP-3, MMP-9, MMP-10, and MMP-13 in OSCC tissue and saliva corroborates the findings of the previous studies [25, 41–46]. Sample inclusion from a small number of OSCC patients is a limitation of this study. A multicenter study with higher patient numbers is recommended to further validate the findings of this study. Since the higher levels of salivary HGF, PIGF, PDGF-BB, MMP-3, MMP-8, and TIMP-2 are first time reported, their role as diagnostic and prognostic markers, and therapeutic targets for OSCC should be thoroughly investigated.

## Conclusions

In conclusion, this study showed overexpression of angiogenic factors HGF, PIGF, and VEGF in OSCC tissues and saliva of OSCC patients. Similarly, among the MMPs tested, the MMP-1, MMP-3, MMP-8, MMP-9, MMP-10, MMP-13, and TIMP-2 were significantly overexpressed in OSCC tissues and saliva of OSCC patients. Our results suggest these overexpressed angiogenic markers and MMPs, especially for HGF and MMP9 as possible saliva-based non-invasive diagnostic, prognostic, and therapeutic biomarkers of OSCC.

## Supplementary Information

**Additional file 1. **Figure S1

**Additional file 2.** Table S1

**Additional file 3.** Table S2 

**Additional file 4. **Table S3 

## Data Availability

The datasets generated and/or analyzed during the current study are available in figshare repository (https://figshare.com/articles/dataset/Raw_data_for_Overexpression_of_angiogenic_factors_and_matrix_metalloproteinases_in_the_saliva_of_oral_squamous_cell_carcinoma_patients_potential_non-invasive_diagnostic_and_therapeutic_biomarkers/19602397).

## Abbreviations

OSCC: Oral squamous cell carcinoma
VEGF: Vascular endothelial growth factor
ANG: Angiogenin
PDGF: Platelet-derived growth factor
HGF: Hepatocyte growth factor
PIGF: Placental growth factor
MMPs: Matrix metalloproteinases
M2BP: Mac-2 binding protein
MRP14: Myeloid-related protein-14
ANT: Adjacent normal tissues
MVD: Microvascular density
HNSCC: Head and neck squamous cell carcinoma
