# Supplementary figures and images for: Overexpression of angiogenic factors and matrix metalloproteinases in the saliva of oral squamous cell carcinoma patients: potential non-invasive diagnostic and therapeutic biomarkers
Source: BMC Cancer. 2022 May 11;22:530. doi: 10.1186/s12885-022-09630-0 (PMC9092712; doi:10.1186/s12885-022-09630-0)

**Supplementary Figure 1**


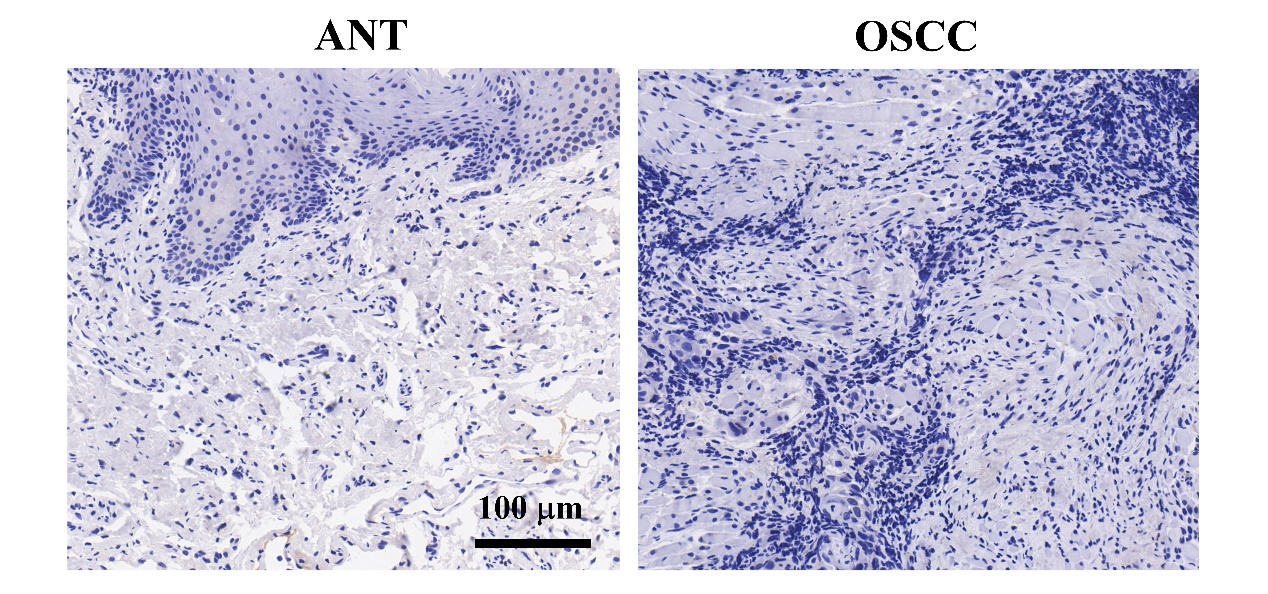


**Figure S1. The negative control of IHC staining of MMP13 for ANT and OSCC.**

Supplement: Supplementary file 1 — Additional file 1. Figure S1 [file 12885_2022_9630_MOESM1_ESM.docx]
