# Supplementary material for: Overexpression of angiogenic factors and matrix metalloproteinases in the saliva of oral squamous cell carcinoma patients: potential non-invasive diagnostic and therapeutic biomarkers
Source: BMC Cancer. 2022 May 11;22:530. doi: 10.1186/s12885-022-09630-0 (PMC9092712; doi:10.1186/s12885-022-09630-0)
Supplement: Supplementary file 2 — Additional file 2. Table S1 [file 12885_2022_9630_MOESM2_ESM.docx]

**Table S1. Volunteer information**

| **No.** | **Sample collection** | **Age** | **Gender** | **TNM stage** | **Anatomical site**  **of the lesion** | **Tumor size (cm)** | **Smoke (Y/N)** |
| --- | --- | --- | --- | --- | --- | --- | --- |
| 1 | Tissue/  Saliva | 42 | Female | T_4_N_0_M_0_ | Right tongue,Mouth floor | 4.0*5.0 | N |
| 2 | Tissue/  Saliva | 63 | Female | T_2_N_0_M_0_ | Left middle of tongue | 3.0*1.5 | N |
| 3 | Tissue/  Saliva | 50 | Female | T_2_N_0_M_0_ | Left margin of tongue | 4.0*3.0 | N |
| 4 | Tissue/  Saliva | 49 | Female | T_3_N_1_M_0_ | Left margin of tongue | 2.7*2.6 | N |
| 5 | Tissue/  Saliva | 74 | Male | T_3_N_2_M_0_ | Left margin of tongue | 4.5*3.0 | N |
| 6 | Tissue/  Saliva | 45 | Male | T_2_N_0_M_0_ | Right margin of tongue | 2.5*1.5 | Y (20 cigarettes per day) |
| 7 | Tissue/  Saliva | 39 | Male | T_1_N_0_M_0_ | Right posterior margin of tongue | 2.0*1.8*1.8 | N |
| 8 | Tissue/  Saliva | 33 | Male | T_3_N_1_M_0_ | Right margin of tongue | 3.0*4.0 | Y (10 cigarettes per day) |
| 9 | Tissue | 74 | Female | T_2_N_0_M_0_ | Right margin of tongue | 3.0*2.0 | N |
| 10 | Tissue | 31 | Male | T_2_N_0_M_0_ | Right tongue,  Mouth floor | 1.3*2.4*2.7 | Y (20 cigarettes per day) |
| 11 | Saliva | 25 | Female | Healthy individual | / | / | N |
| 12 | Saliva | 24 | Female | Healthy individual | / | / | N |
| 13 | Saliva | 23 | Male | Healthy individual | / | / | N |
| 14 | Saliva | 20 | Male | Healthy individual | / | / | N |
| 15 | Saliva | 29 | Female | Healthy individual | / | / | N |
| 16 | Saliva | 29 | Female | Healthy individual | / | / | N |
| 17 | Saliva | 41 | Male | Healthy individual | / | / | N |
| 18 | Saliva | 37 | Male | Healthy individual | / | / | N |

TNM, Tumor Node Metastasis. Y: Yes, N: no.
