# Supplementary material for: Overexpression of angiogenic factors and matrix metalloproteinases in the saliva of oral squamous cell carcinoma patients: potential non-invasive diagnostic and therapeutic biomarkers
Source: BMC Cancer. 2022 May 11;22:530. doi: 10.1186/s12885-022-09630-0 (PMC9092712; doi:10.1186/s12885-022-09630-0)
Supplement: Supplementary file 3 — Additional file 3. Table S2 [file 12885_2022_9630_MOESM3_ESM.docx]

**Table S2.** Primer sequences used fo RT-qPCR analysis

| **Gene** | **Forward 5'->3'** | **Reverse 5'->3'** |
| --- | --- | --- |
| GAPDH | CTCGCTTCGGCAGCACA | AACGCTTCACGAATTTGCGT |
| ANG | CTGGGCGTTTTGTTGTTGGT | GGTTTGGCATCATAGTGCTGG |
| ANGPT2 | ACCCCACTGTTGCTAAAGAAGA | CCATCCTCACGTCGCTGAATA |
| EGF | TGGATGTGCTTGATAAGCGG | ACCATGTCCTTTCCAGTGTGT |
| HGF | GCTATCGGGGTAAAGACCTACA | CGTAGCGTACCTCTGGATTGC |
| PIGF | GAACGGCTCGTCAGAGGTG | ACAGTGCAGATTCTCATCGCC |
| VEGFA | AGGGCAGAATCATCACGAAGT | AGGGTCTCGATTGGATGGCA |
| PDGFB | ATGGAGTTTGCTGTTGAGGTGG | GCAGGGTGGAGGTAGAGAGATG |
| FGF2 | AGTCTTCGCCAGGTCATTGAGATC | CGTCCTGAGTATTCGGCAACAG |
| HBEGF | TCTTCTTCCCTAGCCCCTTGC | GACCCTCCCACTGTATCCACG |
| Lep | CTCCTCCAAACAGAAAGTCACCG | GCTCTTAGAGAAGGCCAGCACGT |
| PECAM1 | GGCTTGTAGTTTTGGCTAGTCCTTG | TGCTTGTTCCACCTTCATTTTCTG |
| KRT18 | CTACAGATGGAGCAGCTCAACGG | GTCCAAGGCATCACCAAGATTAAA |
| MMP-1 | GCCAAGAGCAGATGTGG | GGAGAGTTGTCCCGATGA |
| MMP-2 | CTCCTGGCTCATGCCTTC | GGCGTTCCCATACTTCACA |
| MMP-3 | GGCACAATATGGGCACTT | ATGACCGGCAAGATACAGA |
| MMP-8 | CCCTGGTGCCTTGATGT | GTTTGGGTGTGCTTGGTC |
| MMP-9 | ACGCAGACATCGTCATCC | CCAGGGACCACAACTCG |
| MMP-10 | TGCCCAGCAATACCTAGAA | TGTCACCTCCAACCCAA |
| MMP-13 | GCGACTTCTACCCATTTGA | ACTTGTCCAGGTTTCATCATC |
| TIMP-1 | CTGGCATCCTGTTGTTGCT | GGTGTCCCCACGAACTTG |
| TIMP-2 | CCGTCACAGATGCCAAG | GCAACGACCCTCAAAAGT |

Note: GAPDH (Glyceraldehyde-3-phosphate dehydrogenase), ANG (Angiogenin), ANGPT2 (Angiopoietin 2, ANG-2), EGF (Epidermal growth factor), HGF (Hepatocyte growth factor), PIGF (Placenta growth factor, PIGF), VEGFA (Vascular endothelial growth factor A, VEGF), PDGFB (Platelet-derived growth factor B, PDGF-BB), FGF2 (basic fibroblast growth factor, bFGF), HBEGF (Heparin-binding EGF-like growth factor, HB-EGF), Lep (Leptin), PECAM1 (Platelet endothelial cell adhesion molecule-1, CD31), KRT18 (Cytokeratin 18, CK18).
