# Supplementary material for: Overexpression of angiogenic factors and matrix metalloproteinases in the saliva of oral squamous cell carcinoma patients: potential non-invasive diagnostic and therapeutic biomarkers
Source: BMC Cancer. 2022 May 11;22:530. doi: 10.1186/s12885-022-09630-0 (PMC9092712; doi:10.1186/s12885-022-09630-0)
Supplement: Supplementary file 4 — Additional file 4. Table S3 [file 12885_2022_9630_MOESM4_ESM.docx]

**Table S3 the patients’ information of survival analysis in TCGA database.**

| ID | status | days | location | gender | age | smoke | alcohol | stage |
| --- | --- | --- | --- | --- | --- | --- | --- | --- |
| TCGA.4P.AA8J | alive | 102 | oral tongue | male | 66 | 0 | 0 | 4 |
| TCGA.BA.4074 | dead | 462 | oral tongue | male | 69 | 1 | 1 | 4 |
| TCGA.BA.4075 | dead | 283 | oral tongue | male | 49 | 1 | 1 | 4 |
| TCGA.BA.4076 | dead | 415 | larynx | male | 39 | 1 | 1 | 4 |
| TCGA.BA.4077 | dead | 1134 | base of tongue | female | 45 | 1 | 1 | 4 |
| TCGA.BA.4078 | dead | 276 | larynx | male | 83 | 1 | 0 | 4 |
| TCGA.BA.5149 | alive | 248 | floor of mouth | male | 47 | 1 | 1 | 4 |
| TCGA.BA.5151 | alive | 190 | buccal mucosa | male | 72 | 1 | 1 | 4 |
| TCGA.BA.5152 | alive | 846 | alveolar ridge | male | 56 | 0 | 1 | 4 |
| TCGA.BA.5153 | dead | 1762 | tonsil | male | 51 | 0 | 1 | 3 |
| TCGA.BA.5555 | alive | 186 | larynx | male | 54 | 0 | 1 | 4 |
| TCGA.BA.5556 | alive | 179 | floor of mouth | female | 58 | 1 | 1 | 2 |
| TCGA.BA.5557 | alive | 242 | oral tongue | female | 41 | 0 | 1 | 4 |
| TCGA.BA.5558 | alive | 1636 | hard palate | male | 65 | 0 | 1 | 4 |
| TCGA.BA.5559 | alive | 1747 | tonsil | male | 71 | 0 | 1 | 4 |
| TCGA.BA.6868 | alive | 153 | larynx | male | 53 | 1 | 1 | 4 |
| TCGA.BA.6869 | alive | 244 | larynx | male | 62 | 1 | 1 | 3 |
| TCGA.BA.6870 | dead | 451 | larynx | female | 60 | 1 | 1 | 4 |
| TCGA.BA.6871 | alive | 63 | base of tongue | male | 75 | 1 | 1 | 4 |
| TCGA.BA.6872 | dead | 384 | floor of mouth | male | 47 | 1 | 1 | 4 |
| TCGA.BA.6873 | alive | 113 | oral tongue | male | 28 | 1 | 1 | 4 |
| TCGA.BA.7269 | alive | 236 | oral tongue | male | 61 | 1 | 1 | 3 |
| TCGA.BA.A4IF | alive | 523 | oropharynx | male | 59 | 0 | 1 | 4 |
| TCGA.BA.A4IG | alive | 519 | base of tongue | male | 77 | 0 | 1 | 4 |
| TCGA.BA.A4IH | alive | 201 | tonsil | male | 57 | 0 | 1 | 4 |
| TCGA.BA.A4II | alive | 603 | oropharynx | male | 46 | 0 | 1 | 1 |
| TCGA.BA.A6D8 | alive | 513 | floor of mouth | male | 59 | 0 | 1 | 4 |
| TCGA.BA.A6DA | alive | 351 | larynx | female | 41 | 1 | 1 | 4 |
| TCGA.BA.A6DB | alive | 216 | oral tongue | female | 24 | 0 | 1 | 2 |
| TCGA.BA.A6DD | dead | 173 | floor of mouth | male | 44 | 0 | 1 | 4 |
| TCGA.BA.A6DE | alive | 182 | oral tongue | female | 70 | 0 | 1 | 3 |
| TCGA.BA.A6DG | dead | 69 | oral tongue | male | 49 | 1 | 1 | 4 |
| TCGA.BA.A6DI | dead | 336 | larynx | male | 62 | 0 | 1 | 3 |
| TCGA.BA.A6DJ | alive | 314 | alveolar ridge | male | 62 | 0 | 1 | 4 |
| TCGA.BA.A6DL | alive | 294 | oropharynx | male | 59 | 0 | 1 | 3 |
| TCGA.BA.A8YP | alive | 142 | oropharynx | male | 50 | 1 | 1 | 4 |
| TCGA.BB.4217 | alive | 187 | larynx | male | 68 | 0 | 1 | 4 |
| TCGA.BB.4223 | alive | 2878 | tonsil | male | 48 | 0 | 0 | 4 |
| TCGA.BB.4224 | alive | 278 | oral tongue | male | 52 | 0 | 1 | 3 |
| TCGA.BB.4225 | alive | 140 | base of tongue | male | 73 | 1 | 1 | 4 |
| TCGA.BB.4227 | alive | 133 | hypopharynx | male | 66 | 0 | 1 | 4 |
| TCGA.BB.4228 | alive | 558 | base of tongue | male | 50 | 1 | 1 | 2 |
| TCGA.BB.7861 | alive | 310 | base of tongue | male | 56 | 0 | 0 | 3 |
| TCGA.BB.7862 | alive | 536 | larynx | male | 67 | 0 | 1 | 4 |
| TCGA.BB.7863 | alive | 423 | oral tongue | female | 43 | 0 | 1 | 3 |
| TCGA.BB.7864 | alive | 687 | larynx | male | 61 | 0 | 1 | 4 |
| TCGA.BB.7866 | alive | 638 | tonsil | male | 40 | 0 | 0 | 3 |
| TCGA.BB.7870 | alive | 1153 | larynx | male | 58 | 0 | 1 | 4 |
| TCGA.BB.7871 | alive | 428 | base of tongue | female | 64 | 1 | 1 | 4 |
| TCGA.BB.7872 | alive | 436 | oral tongue | male | 63 | 0 | 1 | 4 |
| TCGA.BB.8596 | alive | 2161 | hypopharynx | female | 69 | 1 | 1 | 4 |
| TCGA.BB.8601 | alive | 624 | floor of mouth | male | 84 | 0 | 1 | 2 |
| TCGA.BB.A5HU | alive | 383 | oral cavity | male | 47 | 0 | 1 | 4 |
| TCGA.BB.A5HY | alive | 308 | hypopharynx | male | 64 | 1 | 1 | 4 |
| TCGA.BB.A5HZ | alive | 350 | oral cavity | male | 65 | 0 | 1 | 4 |
| TCGA.BB.A6UM | alive | 393 | tonsil | male | 52 | 1 | 0 | 3 |
| TCGA.BB.A6UO | dead | 268 | oral tongue | female | 61 | 1 | 0 | 4 |
| TCGA.C9.A47Z | alive | 5 | oral tongue | female | 72 | 0 | 0 | 3 |
| TCGA.C9.A480 | alive | 4 | oral tongue | female | 45 | 0 | 0 | 3 |
| TCGA.CN.4722 | alive | 193 | larynx | female | 61 | 0 | 0 | 2 |
| TCGA.CN.4723 | alive | 412 | larynx | male | 67 | 0 | 1 | 4 |
| TCGA.CN.4725 | alive | 310 | oral tongue | male | 60 | 0 | 0 | 2 |
| TCGA.CN.4726 | dead | 142 | buccal mucosa | male | 68 | 0 | 1 | 4 |
| TCGA.CN.4727 | alive | 194 | larynx | male | 56 | 1 | 1 | 4 |
| TCGA.CN.4728 | alive | 325 | oral cavity | male | 56 | 1 | 1 | 4 |
| TCGA.CN.4729 | alive | 156 | oral cavity | male | 73 | 0 | 1 | 4 |
| TCGA.CN.4730 | alive | 343 | floor of mouth | male | 62 | 1 | 1 | 4 |
| TCGA.CN.4731 | alive | 383 | buccal mucosa | female | 63 | 0 | 1 | 4 |
| TCGA.CN.4733 | alive | 523 | oral tongue | male | 61 | 0 | 0 | 1 |
| TCGA.CN.4734 | alive | 227 | buccal mucosa | male | 70 | 0 | 1 | 2 |
| TCGA.CN.4735 | alive | 369 | larynx | male | 52 | 0 | 1 | 4 |
| TCGA.CN.4736 | dead | 395 | oral tongue | female | 70 | 1 | 1 | 2 |
| TCGA.CN.4737 | alive | 240 | oral tongue | male | 19 | 0 | 0 | 2 |
| TCGA.CN.4738 | dead | 436 | larynx | male | 53 | 1 | 1 | 4 |
| TCGA.CN.4739 | alive | 348 | larynx | male | 71 | 1 | 1 | 4 |
| TCGA.CN.4740 | alive | 811 | oral cavity | female | 79 | 0 | 1 | 4 |
| TCGA.CN.4741 | alive | 876 | alveolar ridge | male | 75 | 1 | 0 | 4 |
| TCGA.CN.4742 | dead | 397 | oral tongue | female | 48 | 1 | 1 | 4 |
| TCGA.CN.5355 | alive | 279 | larynx | male | 64 | 0 | 1 | 4 |
| TCGA.CN.5356 | alive | 289 | larynx | male | 56 | 0 | 0 | 3 |
| TCGA.CN.5358 | alive | 173 | floor of mouth | male | 60 | 1 | 1 | 3 |
| TCGA.CN.5359 | dead | 377 | floor of mouth | male | 59 | 1 | 1 | 4 |
| TCGA.CN.5360 | alive | 867 | larynx | male | 68 | 1 | 1 | 4 |
| TCGA.CN.5361 | alive | 194 | larynx | male | 80 | 1 | 1 | 4 |
| TCGA.CN.5363 | dead | 253 | larynx | male | 48 | 1 | 1 | 4 |
| TCGA.CN.5364 | dead | 493 | floor of mouth | male | 55 | 0 | 1 | 4 |
| TCGA.CN.5365 | dead | 351 | tonsil | male | 38 | 1 | 1 | 4 |
| TCGA.CN.5366 | dead | 360 | hypopharynx | male | 51 | 1 | 1 | 4 |
| TCGA.CN.5367 | dead | 352 | oral tongue | female | 60 | 1 | 1 | 4 |
| TCGA.CN.5369 | dead | 380 | hard palate | female | 90 | 0 | 0 | 4 |
| TCGA.CN.5370 | dead | 259 | oral tongue | male | 78 | 1 | 1 | 2 |
| TCGA.CN.5373 | alive | 413 | floor of mouth | female | 55 | 0 | 1 | 2 |
| TCGA.CN.5374 | alive | 290 | tonsil | female | 56 | 0 | 0 | 4 |
| TCGA.CN.6010 | alive | 207 | larynx | male | 53 | 1 | 1 | 4 |
| TCGA.CN.6011 | alive | 228 | alveolar ridge | male | 57 | 1 | 1 | 4 |
| TCGA.CN.6012 | alive | 144 | larynx | male | 66 | 1 | 0 | 3 |
| TCGA.CN.6013 | alive | 155 | alveolar ridge | male | 56 | 0 | 1 | 4 |
| TCGA.CN.6016 | alive | 202 | floor of mouth | male | 64 | 1 | 1 | 4 |
| TCGA.CN.6017 | alive | 160 | oral tongue | male | 55 | 1 | 1 | 3 |
| TCGA.CN.6018 | alive | 45 | oral cavity | female | 85 | 0 | 0 | 4 |
| TCGA.CN.6019 | alive | 135 | oral tongue | male | 61 | 0 | 1 | 4 |
| TCGA.CN.6020 | alive | 98 | oral cavity | male | 58 | 0 | 1 | 4 |
| TCGA.CN.6021 | dead | 276 | larynx | female | 63 | 1 | 0 | 3 |
| TCGA.CN.6022 | alive | 202 | larynx | male | 49 | 0 | 0 | 4 |
| TCGA.CN.6023 | alive | 188 | larynx | male | 73 | 1 | 1 | 4 |
| TCGA.CN.6024 | alive | 224 | oral tongue | male | 66 | 1 | 1 | 4 |
| TCGA.CN.6988 | alive | 43 | larynx | male | 47 | 0 | 1 | 4 |
| TCGA.CN.6989 | alive | 178 | larynx | male | 64 | 1 | 1 | 4 |
| TCGA.CN.6992 | alive | 83 | larynx | male | 61 | 1 | 1 | 4 |
| TCGA.CN.6994 | alive | 119 | oral cavity | male | 67 | 1 | 0 | 4 |
| TCGA.CN.6995 | dead | 112 | floor of mouth | male | 78 | 0 | 0 | 4 |
| TCGA.CN.6996 | alive | 114 | oral tongue | female | 58 | 0 | 0 | 4 |
| TCGA.CN.6997 | alive | 100 | larynx | male | 66 | 1 | 0 | 4 |
| TCGA.CN.6998 | alive | 46 | oral tongue | male | 53 | 1 | 0 | 4 |
| TCGA.CN.A497 | alive | 490 | larynx | male | 63 | 0 | 0 | 4 |
| TCGA.CN.A498 | alive | 443 | base of tongue | female | 61 | 1 | 0 | 2 |
| TCGA.CN.A499 | alive | 388 | tonsil | female | 60 | 0 | 1 | 1 |
| TCGA.CN.A49A | alive | 391 | alveolar ridge | male | 60 | 0 | 1 | 4 |
| TCGA.CN.A49B | alive | 447 | larynx | male | 71 | 1 | 1 | 3 |
| TCGA.CN.A49C | alive | 190 | tonsil | male | 67 | 1 | 0 | 4 |
| TCGA.CN.A63T | alive | 225 | larynx | male | 60 | 1 | 1 | 4 |
| TCGA.CN.A63U | alive | 526 | larynx | male | 50 | 1 | 0 | 3 |
| TCGA.CN.A63V | alive | 484 | buccal mucosa | male | 59 | 1 | 0 | 4 |
| TCGA.CN.A63W | alive | 348 | larynx | female | 48 | 0 | 1 | 4 |
| TCGA.CN.A641 | alive | 367 | larynx | male | 47 | 1 | 1 | 4 |
| TCGA.CN.A642 | dead | 82 | floor of mouth | male | 57 | 0 | 1 | 4 |
| TCGA.CN.A6UY | alive | 307 | base of tongue | male | 57 | 1 | 0 | 4 |
| TCGA.CN.A6V1 | alive | 223 | tonsil | male | 59 | 0 | 0 | 4 |
| TCGA.CN.A6V3 | alive | 298 | larynx | male | 61 | 1 | 1 | 4 |
| TCGA.CN.A6V6 | alive | 234 | base of tongue | male | 59 | 1 | 1 | 4 |
| TCGA.CN.A6V7 | alive | 188 | tonsil | male | 40 | 0 | 0 | 4 |
| TCGA.CQ.5323 | alive | 1466 | alveolar ridge | male | 82 | 1 | 1 | 3 |
| TCGA.CQ.5324 | alive | 1207 | floor of mouth | male | 59 | 1 | 0 | 4 |
| TCGA.CQ.5325 | dead | 654 | oral tongue | male | 65 | 1 | 1 | 1 |
| TCGA.CQ.5326 | dead | 89 | alveolar ridge | male | 67 | 0 | 1 | 4 |
| TCGA.CQ.5327 | alive | 1184 | oral tongue | female | 61 | 0 | 0 | 4 |
| TCGA.CQ.5329 | alive | 841 | oral tongue | female | 46 | 0 | 0 | 3 |
| TCGA.CQ.5330 | alive | 896 | oral tongue | female | 69 | 1 | 0 | 3 |
| TCGA.CQ.5331 | alive | 1028 | hard palate | female | 73 | 1 | 0 | 4 |
| TCGA.CQ.5332 | dead | 317 | floor of mouth | male | 87 | 0 | 1 | 3 |
| TCGA.CQ.5333 | dead | 341 | oral tongue | male | 74 | 1 | 1 | 1 |
| TCGA.CQ.5334 | dead | 129 | buccal mucosa | male | 87 | 0 | 0 | 4 |
| TCGA.CQ.6218 | alive | 870 | floor of mouth | female | 52 | 1 | 0 | 3 |
| TCGA.CQ.6219 | dead | 479 | oral tongue | female | 50 | 1 | 1 | 4 |
| TCGA.CQ.6220 | dead | 985 | buccal mucosa | male | 69 | 0 | 1 | 3 |
| TCGA.CQ.6221 | alive | 1000 | oral tongue | male | 79 | 1 | 1 | 2 |
| TCGA.CQ.6222 | alive | 1160 | oral tongue | male | 63 | 1 | 0 | 4 |
| TCGA.CQ.6223 | alive | 1057 | alveolar ridge | male | 69 | 1 | 0 | 4 |
| TCGA.CQ.6224 | alive | 985 | oral tongue | male | 52 | 1 | 1 | 2 |
| TCGA.CQ.6225 | dead | 403 | oral tongue | male | 65 | 0 | 0 | 2 |
| TCGA.CQ.6227 | dead | 129 | oral cavity | male | 77 | 1 | 1 | 3 |
| TCGA.CQ.6228 | dead | 456 | floor of mouth | female | 71 | 1 | 1 | 3 |
| TCGA.CQ.6229 | alive | 815 | oral tongue | male | 61 | 0 | 0 | 3 |
| TCGA.CQ.7063 | alive | 1461 | hard palate | female | 59 | 1 | 1 | 1 |
| TCGA.CQ.7065 | alive | 1007 | oral tongue | male | 40 | 0 | 0 | 2 |
| TCGA.CQ.7067 | alive | 509 | oral tongue | female | 75 | 0 | 0 | 1 |
| TCGA.CQ.7068 | alive | 693 | floor of mouth | female | 80 | 0 | 0 | 2 |
| TCGA.CQ.7069 | alive | 901 | alveolar ridge | female | 77 | 0 | 1 | 2 |
| TCGA.CQ.7071 | alive | 877 | oral cavity | female | 76 | 0 | 1 | 4 |
| TCGA.CQ.7072 | alive | 1950 | floor of mouth | male | 51 | 1 | 1 | 4 |
| TCGA.CQ.A4C6 | alive | 968 | buccal mucosa | male | 63 | 1 | 1 | 2 |
| TCGA.CQ.A4C7 | dead | 353 | floor of mouth | male | 88 | 1 | 1 | 2 |
| TCGA.CQ.A4C9 | alive | 613 | floor of mouth | male | 56 | 1 | 0 | 3 |
| TCGA.CQ.A4CB | alive | 545 | floor of mouth | male | 59 | 1 | 1 | 2 |
| TCGA.CQ.A4CD | alive | 548 | oral cavity | male | 69 | 0 | 1 | 4 |
| TCGA.CQ.A4CE | alive | 393 | oral tongue | female | 76 | 0 | 1 | 2 |
| TCGA.CQ.A4CG | alive | 391 | buccal mucosa | female | 78 | 0 | 1 | 2 |
| TCGA.CQ.A4CH | alive | 303 | oral tongue | male | 58 | 0 | 1 | 4 |
| TCGA.CQ.A4CI | alive | 608 | buccal mucosa | male | 73 | 1 | 1 | 3 |
| TCGA.CR.5243 | alive | 2562 | tonsil | male | 51 | 0 | 1 | 4 |
| TCGA.CR.5247 | alive | 358 | tonsil | male | 48 | 1 | 1 | 3 |
| TCGA.CR.5248 | alive | 1663 | tonsil | male | 53 | 1 | 1 | 4 |
| TCGA.CR.5249 | alive | 1152 | tonsil | female | 35 | 0 | 1 | 2 |
| TCGA.CR.5250 | alive | 799 | base of tongue | male | 71 | 1 | 1 | 2 |
| TCGA.CR.6467 | alive | 1777 | tonsil | male | 59 | 1 | 1 | 4 |
| TCGA.CR.6470 | alive | 1521 | tonsil | male | 38 | 0 | 1 | 4 |
| TCGA.CR.6471 | dead | 1202 | oral cavity | male | 58 | 1 | 1 | 4 |
| TCGA.CR.6472 | alive | 1050 | base of tongue | male | 59 | 0 | 1 | 4 |
| TCGA.CR.6473 | alive | 1125 | hypopharynx | male | 68 | 1 | 1 | 4 |
| TCGA.CR.6474 | dead | 564 | larynx | male | 51 | 1 | 1 | 4 |
| TCGA.CR.6477 | alive | 514 | base of tongue | female | 56 | 1 | 1 | 4 |
| TCGA.CR.6478 | dead | 183 | tonsil | female | 66 | 1 | 1 | 4 |
| TCGA.CR.6480 | alive | 362 | tonsil | male | 53 | 0 | 1 | 4 |
| TCGA.CR.6481 | alive | 311 | tonsil | male | 47 | 0 | 1 | 4 |
| TCGA.CR.6482 | alive | 345 | tonsil | male | 62 | 1 | 1 | 4 |
| TCGA.CR.6484 | alive | 354 | oral cavity | female | 67 | 0 | 0 | 4 |
| TCGA.CR.6487 | alive | 234 | tonsil | male | 50 | 1 | 1 | 2 |
| TCGA.CR.6488 | alive | 379 | oral tongue | female | 68 | 0 | 1 | 2 |
| TCGA.CR.6491 | alive | 350 | floor of mouth | male | 60 | 1 | 1 | 4 |
| TCGA.CR.6492 | alive | 479 | hard palate | male | 78 | 1 | 1 | 4 |
| TCGA.CR.6493 | dead | 282 | oral tongue | male | 69 | 1 | 1 | 4 |
| TCGA.CR.7364 | alive | 1435 | larynx | male | 66 | 1 | 0 | 3 |
| TCGA.CR.7365 | alive | 1191 | oral cavity | male | 60 | 1 | 1 | 4 |
| TCGA.CR.7367 | alive | 1440 | oral cavity | male | 52 | 1 | 1 | 4 |
| TCGA.CR.7368 | alive | 1245 | oral cavity | male | 54 | 1 | 1 | 4 |
| TCGA.CR.7369 | dead | 1090 | oral cavity | male | 59 | 1 | 1 | 4 |
| TCGA.CR.7370 | alive | 105 | larynx | female | 72 | 1 | 1 | 2 |
| TCGA.CR.7371 | dead | 94 | larynx | female | 45 | 1 | 0 | 3 |
| TCGA.CR.7372 | alive | 759 | oral tongue | male | 45 | 0 | 1 | 2 |
| TCGA.CR.7373 | alive | 889 | oral cavity | male | 66 | 1 | 0 | 4 |
| TCGA.CR.7374 | alive | 30 | larynx | female | 67 | 1 | 1 | 2 |
| TCGA.CR.7376 | alive | 972 | oral cavity | male | 83 | 1 | 1 | 2 |
| TCGA.CR.7377 | dead | 279 | oral cavity | male | 58 | 1 | 1 | 4 |
| TCGA.CR.7379 | alive | 1036 | oral cavity | female | 78 | 0 | 0 | 4 |
| TCGA.CR.7380 | dead | 606 | oral cavity | male | 58 | 0 | 1 | 3 |
| TCGA.CR.7382 | alive | 796 | oral tongue | male | 49 | 0 | 1 | 4 |
| TCGA.CR.7383 | dead | 521 | tonsil | female | 79 | 1 | 1 | 1 |
| TCGA.CR.7385 | alive | 997 | tonsil | male | 42 | 1 | 1 | 4 |
| TCGA.CR.7386 | alive | 1430 | oral cavity | male | 69 | 1 | 1 | 4 |
| TCGA.CR.7388 | dead | 823 | larynx | female | 70 | 1 | 1 | 4 |
| TCGA.CR.7389 | alive | 392 | larynx | male | 55 | 1 | 1 | 3 |
| TCGA.CR.7390 | alive | 907 | oral tongue | male | 67 | 1 | 1 | 3 |
| TCGA.CR.7391 | alive | 913 | oral tongue | female | 36 | 1 | 1 | 1 |
| TCGA.CR.7392 | alive | 946 | oral tongue | female | 67 | 1 | 0 | 4 |
| TCGA.CR.7393 | alive | 908 | oral tongue | male | 26 | 0 | 1 | 1 |
| TCGA.CR.7394 | alive | 898 | oral tongue | male | 70 | 1 | 0 | 4 |
| TCGA.CR.7395 | alive | 930 | oral cavity | female | 80 | 1 | 1 | 2 |
| TCGA.CR.7397 | alive | 216 | oral tongue | male | 44 | 1 | 1 | 4 |
| TCGA.CR.7398 | alive | 156 | larynx | female | 53 | 0 | 1 | 2 |
| TCGA.CR.7399 | alive | 181 | larynx | female | 60 | 1 | 1 | 4 |
| TCGA.CR.7401 | alive | 1077 | oral tongue | male | 64 | 1 | 1 | 1 |
| TCGA.CR.7402 | alive | 911 | larynx | male | 68 | 1 | 1 | 3 |
| TCGA.CR.7404 | alive | 1472 | tonsil | male | 53 | 0 | 1 | 4 |
| TCGA.CV.5430 | alive | 4115 | larynx | male | 61 | 1 | 1 | 4 |
| TCGA.CV.5431 | dead | 522 | larynx | male | 73 | 1 | 1 | 4 |
| TCGA.CV.5432 | alive | 3930 | larynx | male | 68 | 1 | 0 | 3 |
| TCGA.CV.5434 | dead | 3314 | larynx | male | 60 | 0 | 0 | 4 |
| TCGA.CV.5435 | dead | 2319 | larynx | male | 57 | 1 | 1 | 4 |
| TCGA.CV.5436 | dead | 584 | floor of mouth | male | 65 | 1 | 1 | 4 |
| TCGA.CV.5439 | dead | 546 | base of tongue | male | 62 | 0 | 1 | 2 |
| TCGA.CV.5440 | alive | 2732 | larynx | male | 52 | 1 | 1 | 4 |
| TCGA.CV.5441 | alive | 2663 | larynx | male | 58 | 0 | 1 | 4 |
| TCGA.CV.5442 | alive | 2327 | hard palate | female | 76 | 0 | 0 | 4 |
| TCGA.CV.5443 | alive | 2567 | larynx | male | 63 | 1 | 1 | 3 |
| TCGA.CV.5444 | alive | 1969 | larynx | male | 64 | 0 | 1 | 4 |
| TCGA.CV.5966 | dead | 545 | oral cavity | female | 63 | 1 | 1 | 4 |
| TCGA.CV.5970 | dead | 406 | oral tongue | male | 59 | 1 | 1 | 4 |
| TCGA.CV.5971 | alive | 540 | oral tongue | male | 60 | 0 | 1 | 4 |
| TCGA.CV.5973 | alive | 1737 | oral tongue | female | 62 | 0 | 0 | 3 |
| TCGA.CV.5976 | alive | 1478 | oral tongue | male | 50 | 1 | 1 | 3 |
| TCGA.CV.5977 | alive | 1007 | oral tongue | male | 66 | 0 | 1 | 3 |
| TCGA.CV.5978 | dead | 215 | larynx | female | 53 | 0 | 1 | 4 |
| TCGA.CV.5979 | alive | 1315 | oral tongue | male | 26 | 0 | 1 | 3 |
| TCGA.CV.6003 | alive | 1246 | oral tongue | female | 50 | 0 | 1 | 2 |
| TCGA.CV.6433 | alive | 405 | oral tongue | male | 57 | 1 | 1 | 2 |
| TCGA.CV.6436 | alive | 743 | oral tongue | male | 62 | 1 | 0 | 3 |
| TCGA.CV.6441 | dead | 292 | oral tongue | male | 60 | 1 | 1 | 3 |
| TCGA.CV.6933 | dead | 2741 | oral tongue | male | 53 | 0 | 1 | 4 |
| TCGA.CV.6934 | dead | 65 | oral tongue | female | 66 | 1 | 0 | 4 |
| TCGA.CV.6935 | dead | 295 | larynx | male | 67 | 1 | 1 | 3 |
| TCGA.CV.6936 | dead | 166 | floor of mouth | male | 68 | 1 | 1 | 4 |
| TCGA.CV.6937 | dead | 624 | oral cavity | male | 71 | 0 | 0 | 2 |
| TCGA.CV.6938 | dead | 144 | oral cavity | male | 87 | 0 | 0 | 2 |
| TCGA.CV.6939 | dead | 666 | oral tongue | male | 60 | 0 | 0 | 4 |
| TCGA.CV.6940 | dead | 804 | buccal mucosa | female | 80 | 1 | 0 | 1 |
| TCGA.CV.6941 | dead | 342 | oral tongue | male | 51 | 1 | 1 | 3 |
| TCGA.CV.6942 | alive | 3835 | oral cavity | female | 73 | 0 | 0 | 2 |
| TCGA.CV.6943 | dead | 602 | base of tongue | male | 74 | 1 | 1 | 3 |
| TCGA.CV.6945 | dead | 366 | oral tongue | male | 41 | 1 | 1 | 4 |
| TCGA.CV.6948 | dead | 1289 | floor of mouth | female | 79 | 0 | 0 | 4 |
| TCGA.CV.6950 | dead | 459 | base of tongue | male | 64 | 0 | 1 | 4 |
| TCGA.CV.6951 | dead | 915 | oral tongue | male | 57 | 0 | 1 | 4 |
| TCGA.CV.6952 | dead | 185 | oral tongue | female | 65 | 1 | 1 | 3 |
| TCGA.CV.6953 | dead | 1641 | floor of mouth | female | 80 | 0 | 0 | 3 |
| TCGA.CV.6954 | dead | 2002 | oral tongue | male | 59 | 1 | 1 | 4 |
| TCGA.CV.6955 | dead | 334 | oral cavity | female | 74 | 0 | 0 | 2 |
| TCGA.CV.6956 | dead | 217 | oral tongue | male | 67 | 0 | 1 | 4 |
| TCGA.CV.6959 | dead | 256 | oral tongue | male | 48 | 0 | 1 | 3 |
| TCGA.CV.6960 | dead | 862 | oral cavity | male | 49 | 1 | 1 | 3 |
| TCGA.CV.6961 | dead | 76 | oral tongue | male | 61 | 1 | 1 | 2 |
| TCGA.CV.6962 | dead | 126 | larynx | male | 65 | 0 | 0 | 3 |
| TCGA.CV.7089 | dead | 1972 | larynx | male | 74 | 0 | 1 | 4 |
| TCGA.CV.7090 | alive | 3837 | oral cavity | male | 39 | 0 | 0 | 2 |
| TCGA.CV.7091 | alive | 3381 | oral cavity | male | 54 | 1 | 1 | 1 |
| TCGA.CV.7095 | dead | 572 | oral cavity | female | 87 | 1 | 0 | 4 |
| TCGA.CV.7097 | dead | 385 | oral cavity | male | 53 | 1 | 1 | 3 |
| TCGA.CV.7099 | dead | 243 | oral cavity | female | 85 | 1 | 0 | 2 |
| TCGA.CV.7100 | dead | 274 | oral cavity | male | 66 | 1 | 0 | 2 |
| TCGA.CV.7101 | dead | 160 | larynx | male | 80 | 1 | 1 | 2 |
| TCGA.CV.7102 | dead | 56 | floor of mouth | female | 76 | 1 | 1 | 3 |
| TCGA.CV.7103 | dead | 1591 | oral tongue | male | 49 | 0 | 1 | 2 |
| TCGA.CV.7104 | dead | 393 | oral tongue | female | 61 | 0 | 0 | 4 |
| TCGA.CV.7177 | dead | 663 | larynx | female | 82 | 0 | 0 | 1 |
| TCGA.CV.7178 | dead | 2166 | oral cavity | female | 64 | 0 | 1 | 4 |
| TCGA.CV.7180 | dead | 327 | oral tongue | male | 34 | 1 | 1 | 2 |
| TCGA.CV.7183 | alive | 3497 | oral cavity | male | 53 | 1 | 0 | 2 |
| TCGA.CV.7235 | alive | 1724 | floor of mouth | male | 67 | 1 | 1 | 3 |
| TCGA.CV.7236 | dead | 144 | oral tongue | female | 77 | 0 | 0 | 4 |
| TCGA.CV.7238 | alive | 1444 | oral tongue | female | 69 | 0 | 0 | 2 |
| TCGA.CV.7242 | alive | 1095 | larynx | female | 60 | 1 | 1 | 3 |
| TCGA.CV.7243 | alive | 954 | oral tongue | male | 50 | 0 | 1 | 2 |
| TCGA.CV.7245 | alive | 797 | larynx | male | 62 | 1 | 0 | 3 |
| TCGA.CV.7247 | dead | 577 | larynx | male | 55 | 0 | 0 | 2 |
| TCGA.CV.7248 | dead | 521 | larynx | female | 63 | 1 | 0 | 4 |
| TCGA.CV.7250 | dead | 2900 | larynx | male | 64 | 1 | 1 | 3 |
| TCGA.CV.7252 | dead | 151 | oral cavity | female | 62 | 0 | 0 | 3 |
| TCGA.CV.7253 | dead | 361 | oral cavity | male | 58 | 0 | 0 | 2 |
| TCGA.CV.7254 | dead | 1459 | oral cavity | male | 55 | 1 | 1 | 2 |
| TCGA.CV.7255 | dead | 64 | oral tongue | female | 32 | 0 | 1 | 2 |
| TCGA.CV.7261 | alive | 1099 | larynx | male | 57 | 1 | 0 | 3 |
| TCGA.CV.7263 | dead | 560 | oral cavity | male | 64 | 0 | 0 | 2 |
| TCGA.CV.7406 | dead | 1748 | base of tongue | male | 49 | 1 | 1 | 2 |
| TCGA.CV.7407 | dead | 1081 | floor of mouth | female | 67 | 0 | 0 | 2 |
| TCGA.CV.7409 | dead | 543 | oral cavity | male | 43 | 0 | 0 | 4 |
| TCGA.CV.7410 | dead | 6417 | larynx | male | 61 | 1 | 0 | 2 |
| TCGA.CV.7411 | dead | 2717 | oral cavity | female | 64 | 0 | 0 | 4 |
| TCGA.CV.7413 | dead | 294 | oral cavity | female | 74 | 0 | 0 | 2 |
| TCGA.CV.7414 | dead | 14 | oral cavity | male | 78 | 0 | 0 | 3 |
| TCGA.CV.7415 | dead | 695 | larynx | male | 60 | 0 | 0 | 3 |
| TCGA.CV.7416 | dead | 763 | oral cavity | female | 29 | 0 | 0 | 4 |
| TCGA.CV.7418 | dead | 789 | larynx | male | 62 | 1 | 1 | 4 |
| TCGA.CV.7421 | dead | 2 | larynx | male | 76 | 0 | 0 | 4 |
| TCGA.CV.7422 | dead | 1037 | larynx | female | 60 | 1 | 1 | 4 |
| TCGA.CV.7423 | dead | 3059 | oral cavity | male | 65 | 0 | 0 | 2 |
| TCGA.CV.7424 | dead | 453 | larynx | male | 67 | 1 | 0 | 4 |
| TCGA.CV.7425 | dead | 1718 | oral cavity | female | 77 | 0 | 0 | 3 |
| TCGA.CV.7427 | dead | 4760 | oral cavity | female | 73 | 1 | 1 | 2 |
| TCGA.CV.7428 | dead | 1671 | oral cavity | male | 47 | 1 | 1 | 4 |
| TCGA.CV.7429 | dead | 107 | oral cavity | male | 55 | 0 | 1 | 3 |
| TCGA.CV.7430 | dead | 495 | larynx | male | 56 | 1 | 0 | 3 |
| TCGA.CV.7432 | dead | 2570 | oral cavity | male | 79 | 1 | 0 | 3 |
| TCGA.CV.7433 | dead | 601 | larynx | male | 49 | 1 | 0 | 4 |
| TCGA.CV.7434 | dead | 218 | oral cavity | male | 64 | 1 | 1 | 3 |
| TCGA.CV.7435 | alive | 4335 | oral cavity | female | 57 | 1 | 0 | 4 |
| TCGA.CV.7437 | dead | 506 | larynx | male | 77 | 0 | 0 | 2 |
| TCGA.CV.7438 | dead | 194 | oral tongue | female | 87 | 0 | 0 | 2 |
| TCGA.CV.7440 | dead | 675 | larynx | male | 38 | 1 | 0 | 2 |
| TCGA.CV.7446 | dead | 1093 | oral tongue | male | 66 | 1 | 1 | 2 |
| TCGA.CV.7568 | dead | 927 | oral cavity | female | 48 | 1 | 0 | 4 |
| TCGA.CV.A45O | alive | 851 | alveolar ridge | male | 57 | 0 | 0 | 3 |
| TCGA.CV.A45P | alive | 639 | oral tongue | female | 82 | 0 | 0 | 2 |
| TCGA.CV.A45Q | dead | 5152 | oral cavity | female | 69 | 1 | 0 | 4 |
| TCGA.CV.A45R | alive | 5480 | oral tongue | male | 46 | 0 | 1 | 3 |
| TCGA.CV.A45T | dead | 4856 | oral tongue | female | 64 | 0 | 1 | 2 |
| TCGA.CV.A45U | dead | 1079 | oral cavity | male | 59 | 0 | 1 | 4 |
| TCGA.CV.A45V | dead | 32 | oral cavity | female | 87 | 0 | 0 | 4 |
| TCGA.CV.A45W | dead | 1398 | larynx | male | 75 | 0 | 1 | 3 |
| TCGA.CV.A45X | dead | 198 | floor of mouth | male | 47 | 0 | 1 | 4 |
| TCGA.CV.A45Y | dead | 2703 | larynx | male | 61 | 1 | 1 | 4 |
| TCGA.CV.A45Z | dead | 1466 | larynx | male | 75 | 1 | 1 | 2 |
| TCGA.CV.A460 | dead | 1838 | larynx | male | 72 | 1 | 0 | 4 |
| TCGA.CV.A461 | dead | 2064 | larynx | male | 65 | 1 | 0 | 3 |
| TCGA.CV.A463 | dead | 23 | floor of mouth | female | 82 | 0 | 0 | 4 |
| TCGA.CV.A464 | alive | 1722 | buccal mucosa | male | 48 | 1 | 1 | 4 |
| TCGA.CV.A465 | dead | 215 | oral tongue | male | 24 | 0 | 0 | 2 |
| TCGA.CV.A468 | dead | 464 | lip | male | 42 | 1 | 1 | 3 |
| TCGA.CV.A6JD | dead | 182 | floor of mouth | female | 82 | 1 | 0 | 4 |
| TCGA.CV.A6JE | alive | 750 | oral cavity | male | 78 | 1 | 1 | 2 |
| TCGA.CV.A6JM | dead | 194 | hypopharynx | male | 85 | 0 | 0 | 4 |
| TCGA.CV.A6JN | alive | 717 | oral cavity | male | 53 | 1 | 1 | 2 |
| TCGA.CV.A6JO | dead | 197 | oral tongue | male | 69 | 1 | 0 | 4 |
| TCGA.CV.A6JT | alive | 670 | oral tongue | male | 65 | 0 | 0 | 2 |
| TCGA.CV.A6JU | alive | 110 | oral tongue | female | 61 | 1 | 1 | 4 |
| TCGA.CV.A6JY | alive | 646 | oral cavity | male | 69 | 1 | 1 | 4 |
| TCGA.CV.A6JZ | alive | 680 | oral cavity | male | 68 | 1 | 1 | 4 |
| TCGA.CV.A6K0 | alive | 606 | oral tongue | male | 58 | 0 | 1 | 2 |
| TCGA.CV.A6K1 | alive | 527 | larynx | male | 65 | 1 | 1 | 4 |
| TCGA.CV.A6K2 | dead | 317 | oral cavity | male | 79 | 1 | 1 | 2 |
| TCGA.CX.7082 | dead | 11 | oral cavity | male | 82 | 0 | 1 | 2 |
| TCGA.CX.7085 | alive | 15 | oral tongue | female | 77 | 0 | 1 | 3 |
| TCGA.CX.7086 | alive | 237 | floor of mouth | male | 53 | 1 | 1 | 4 |
| TCGA.CX.7219 | alive | 828 | floor of mouth | male | 47 | 1 | 1 | 4 |
| TCGA.CX.A4AQ | alive | 1555 | floor of mouth | male | 56 | 0 | 1 | 4 |
| TCGA.D6.6515 | dead | 403 | oral tongue | female | 82 | 0 | 0 | 2 |
| TCGA.D6.6516 | alive | 325 | lip | male | 69 | 1 | 0 | 3 |
| TCGA.D6.6517 | alive | 250 | larynx | male | 59 | 1 | 1 | 3 |
| TCGA.D6.6823 | alive | 605 | oral tongue | male | 50 | 1 | 1 | 3 |
| TCGA.D6.6824 | alive | 77 | larynx | male | 61 | 1 | 1 | 4 |
| TCGA.D6.6825 | alive | 153 | oral tongue | male | 73 | 1 | 1 | 3 |
| TCGA.D6.6826 | alive | 154 | larynx | female | 64 | 0 | 0 | 4 |
| TCGA.D6.6827 | alive | 128 | lip | female | 55 | 1 | 0 | 3 |
| TCGA.D6.8568 | alive | 106 | larynx | male | 62 | 1 | 1 | 2 |
| TCGA.D6.8569 | alive | 128 | oral tongue | male | 52 | 1 | 0 | 2 |
| TCGA.D6.A4Z9 | alive | 112 | oral tongue | male | 59 | 1 | 0 | 4 |
| TCGA.D6.A4ZB | alive | 104 | oral tongue | male | 61 | 1 | 0 | 3 |
| TCGA.D6.A6EK | alive | 483 | larynx | male | 67 | 1 | 0 | 4 |
| TCGA.D6.A6EM | alive | 232 | oral tongue | female | 65 | 0 | 0 | 3 |
| TCGA.D6.A6EN | alive | 439 | buccal mucosa | male | 71 | 0 | 0 | 3 |
| TCGA.D6.A6EO | alive | 429 | floor of mouth | male | 44 | 0 | 0 | 4 |
| TCGA.D6.A6EP | alive | 424 | hypopharynx | male | 62 | 1 | 0 | 3 |
| TCGA.D6.A6EQ | alive | 368 | larynx | male | 57 | 0 | 0 | 4 |
| TCGA.D6.A6ES | alive | 24 | larynx | male | 50 | 0 | 0 | 4 |
| TCGA.D6.A74Q | alive | 338 | larynx | male | 67 | 1 | 0 | 4 |
| TCGA.DQ.5624 | alive | 1149 | oral tongue | female | 43 | 0 | 0 | 4 |
| TCGA.DQ.5625 | alive | 1058 | oral tongue | female | 52 | 1 | 1 | 2 |
| TCGA.DQ.5629 | alive | 516 | larynx | male | 64 | 1 | 1 | 4 |
| TCGA.DQ.5630 | alive | 477 | oral tongue | male | 73 | 1 | 1 | 3 |
| TCGA.DQ.5631 | dead | 548 | oral tongue | male | 52 | 1 | 1 | 4 |
| TCGA.DQ.7588 | dead | 427 | buccal mucosa | male | 66 | 1 | 1 | 3 |
| TCGA.DQ.7589 | alive | 575 | larynx | male | 70 | 1 | 1 | 4 |
| TCGA.DQ.7590 | alive | 661 | tonsil | male | 51 | 1 | 1 | 4 |
| TCGA.DQ.7591 | alive | 360 | base of tongue | male | 62 | 1 | 1 | 4 |
| TCGA.DQ.7592 | alive | 299 | oral tongue | male | 57 | 1 | 1 | 4 |
| TCGA.DQ.7593 | alive | 369 | base of tongue | male | 58 | 1 | 1 | 4 |
| TCGA.DQ.7594 | alive | 368 | base of tongue | male | 47 | 0 | 1 | 4 |
| TCGA.DQ.7595 | alive | 324 | larynx | male | 53 | 1 | 1 | 2 |
| TCGA.DQ.7596 | alive | 340 | tonsil | male | 48 | 0 | 1 | 4 |
| TCGA.F7.7848 | alive | 35 | larynx | male | 47 | 1 | 1 | 4 |
| TCGA.F7.8298 | alive | 14 | larynx | male | 58 | 1 | 1 | 1 |
| TCGA.F7.8489 | alive | 14 | floor of mouth | male | 48 | 1 | 0 | 2 |
| TCGA.F7.A50G | alive | 15 | oral tongue | male | 66 | 1 | 0 | 3 |
| TCGA.F7.A50I | alive | 92 | larynx | male | 72 | 1 | 1 | 4 |
| TCGA.F7.A50J | alive | 156 | oral tongue | female | 67 | 1 | 1 | 3 |
| TCGA.F7.A61S | alive | 3 | oral tongue | male | 62 | 1 | 1 | 3 |
| TCGA.F7.A61V | alive | 9 | base of tongue | male | 54 | 1 | 0 | 2 |
| TCGA.F7.A61W | alive | 14 | oral tongue | male | 51 | 1 | 1 | 4 |
| TCGA.F7.A620 | alive | 13 | base of tongue | male | 47 | 1 | 1 | 3 |
| TCGA.F7.A622 | alive | 13 | larynx | male | 75 | 0 | 1 | 3 |
| TCGA.F7.A623 | alive | 6 | larynx | male | 70 | 1 | 1 | 4 |
| TCGA.F7.A624 | alive | 0 | buccal mucosa | male | 73 | 0 | 1 | 2 |
| TCGA.H7.7774 | alive | 30 | oral cavity | female | 75 | 1 | 1 | 3 |
| TCGA.H7.8501 | alive | 58 | buccal mucosa | male | 54 | 0 | 1 | 4 |
| TCGA.H7.8502 | alive | 133 | oral cavity | male | 50 | 1 | 1 | 4 |
| TCGA.H7.A6C4 | alive | 414 | oral tongue | female | 35 | 1 | 1 | 3 |
| TCGA.H7.A76A | alive | 206 | tonsil | male | 57 | 1 | 1 | 4 |
| TCGA.HD.7229 | alive | 2 | larynx | male | 60 | 0 | 0 | 4 |
| TCGA.HD.7753 | alive | 5 | oropharynx | male | 62 | 0 | 1 | 2 |
| TCGA.HD.7754 | alive | 25 | tonsil | male | 69 | 0 | 1 | 4 |
| TCGA.HD.7831 | alive | 0 | oral tongue | male | 74 | 0 | 1 | 3 |
| TCGA.HD.7832 | alive | 0 | floor of mouth | male | 52 | 0 | 1 | 4 |
| TCGA.HD.7917 | alive | 37 | floor of mouth | male | 62 | 0 | 1 | 2 |
| TCGA.HD.8224 | alive | 55 | base of tongue | male | 63 | 0 | 1 | 3 |
| TCGA.HD.8314 | alive | 44 | base of tongue | male | 58 | 0 | 0 | 3 |
| TCGA.HD.8634 | alive | 24 | oral tongue | female | 51 | 1 | 1 | 1 |
| TCGA.HD.8635 | alive | 25 | oral tongue | female | 61 | 1 | 1 | 3 |
| TCGA.HD.A4C1 | alive | 11 | buccal mucosa | female | 41 | 0 | 0 | 4 |
| TCGA.HD.A633 | alive | 49 | oral cavity | male | 74 | 0 | 1 | 4 |
| TCGA.HD.A634 | alive | 10 | tonsil | male | 56 | 1 | 1 | 3 |
| TCGA.HD.A6HZ | alive | 111 | oral tongue | female | 79 | 0 | 0 | 2 |
| TCGA.HD.A6I0 | alive | 33 | oral cavity | male | 56 | 1 | 1 | 3 |
| TCGA.HL.7533 | alive | 692 | oral cavity | male | 65 | 1 | 1 | 4 |
| TCGA.IQ.7630 | alive | 160 | oropharynx | male | 49 | 0 | 0 | 3 |
| TCGA.IQ.7631 | alive | 122 | buccal mucosa | female | 60 | 1 | 0 | 2 |
| TCGA.IQ.7632 | alive | 113 | alveolar ridge | female | 68 | 1 | 0 | 4 |
| TCGA.IQ.A61E | alive | 588 | oral tongue | female | 55 | 1 | 1 | 3 |
| TCGA.IQ.A61G | alive | 360 | floor of mouth | male | 57 | 1 | 0 | 4 |
| TCGA.IQ.A61H | alive | 412 | oral tongue | male | 76 | 0 | 0 | 2 |
| TCGA.IQ.A61I | dead | 2 | oropharynx | male | 63 | 0 | 0 | 4 |
| TCGA.IQ.A61J | alive | 440 | oral tongue | male | 54 | 0 | 0 | 4 |
| TCGA.IQ.A61O | alive | 360 | oropharynx | male | 43 | 0 | 0 | 4 |
| TCGA.IQ.A6SG | alive | 579 | oral tongue | female | 61 | 0 | 0 | 3 |
| TCGA.IQ.A6SH | alive | 471 | oral tongue | male | 55 | 1 | 0 | 4 |
| TCGA.KU.A66S | alive | 224 | larynx | female | 69 | 1 | 0 | 3 |
| TCGA.KU.A66T | alive | 210 | floor of mouth | female | 53 | 0 | 0 | 4 |
| TCGA.KU.A6H7 | alive | 146 | tonsil | female | 55 | 1 | 1 | 4 |
| TCGA.KU.A6H8 | alive | 168 | oral tongue | male | 41 | 1 | 1 | 1 |
| TCGA.MT.A51W | alive | 14 | tonsil | female | 52 | 1 | 1 | 1 |
| TCGA.MT.A51X | alive | 34 | oral tongue | male | 30 | 1 | 0 | 4 |
| TCGA.MT.A67A | alive | 392 | oral tongue | female | 85 | 0 | 1 | 1 |
| TCGA.MT.A67D | alive | 56 | floor of mouth | male | 55 | 1 | 1 | 2 |
| TCGA.MT.A67F | alive | 111 | oral cavity | female | 60 | 0 | 1 | 4 |
| TCGA.MT.A7BN | alive | 51 | floor of mouth | male | 74 | 0 | 1 | 4 |
| TCGA.MZ.A5BI | dead | 217 | tonsil | male | 53 | 0 | 1 | 4 |
| TCGA.MZ.A6I9 | alive | 254 | base of tongue | male | 68 | 1 | 1 | 4 |
| TCGA.MZ.A7D7 | alive | 240 | base of tongue | male | 51 | 1 | 1 | 4 |
| TCGA.P3.A5Q5 | alive | 910 | tonsil | male | 54 | 0 | 1 | 4 |
| TCGA.P3.A5Q6 | dead | 480 | tonsil | male | 49 | 0 | 1 | 3 |
| TCGA.P3.A5QA | alive | 1652 | oral tongue | male | 41 | 0 | 1 | 2 |
| TCGA.P3.A5QE | alive | 721 | base of tongue | male | 49 | 0 | 1 | 3 |
| TCGA.P3.A5QF | dead | 330 | alveolar ridge | male | 49 | 0 | 0 | 4 |
| TCGA.P3.A6SW | alive | 1120 | tonsil | male | 50 | 0 | 1 | 4 |
| TCGA.P3.A6SX | dead | 1430 | tonsil | male | 67 | 1 | 1 | 4 |
| TCGA.P3.A6T0 | alive | 578 | floor of mouth | female | 47 | 1 | 1 | 4 |
| TCGA.P3.A6T2 | alive | 1917 | buccal mucosa | male | 45 | 0 | 1 | 2 |
| TCGA.P3.A6T3 | dead | 577 | oral cavity | male | 49 | 0 | 1 | 4 |
| TCGA.P3.A6T4 | dead | 62 | floor of mouth | male | 54 | 1 | 1 | 4 |
| TCGA.P3.A6T5 | dead | 882 | alveolar ridge | female | 79 | 0 | 1 | 4 |
| TCGA.P3.A6T6 | dead | 395 | alveolar ridge | male | 53 | 0 | 1 | 4 |
| TCGA.P3.A6T7 | dead | 487 | floor of mouth | male | 55 | 0 | 1 | 3 |
| TCGA.P3.A6T8 | alive | 400 | floor of mouth | male | 54 | 1 | 1 | 4 |
| TCGA.QK.A64Z | alive | 553 | hard palate | female | 79 | 0 | 1 | 2 |
| TCGA.QK.A652 | alive | 259 | oral tongue | male | 60 | 0 | 1 | 2 |
| TCGA.QK.A6IF | alive | 245 | tonsil | male | 61 | 0 | 1 | 4 |
| TCGA.QK.A6IG | alive | 218 | buccal mucosa | male | 69 | 1 | 1 | 2 |
| TCGA.QK.A6IH | alive | 228 | alveolar ridge | female | 65 | 0 | 1 | 4 |
| TCGA.QK.A6II | alive | 225 | floor of mouth | male | 52 | 1 | 1 | 3 |
| TCGA.QK.A6IJ | alive | 64 | floor of mouth | male | 71 | 1 | 1 | 2 |
| TCGA.QK.A6V9 | alive | 567 | tonsil | male | 56 | 0 | 0 | 2 |
| TCGA.QK.A6VB | alive | 156 | floor of mouth | male | 66 | 0 | 0 | 4 |
| TCGA.QK.A6VC | alive | 106 | hypopharynx | female | 62 | 0 | 0 | 4 |
| TCGA.QK.A8Z7 | alive | 23 | floor of mouth | male | 59 | 1 | 1 | 4 |
| TCGA.QK.A8Z8 | dead | 171 | larynx | female | 60 | 0 | 1 | 4 |
| TCGA.QK.A8Z9 | alive | 149 | floor of mouth | male | 56 | 0 | 1 | 4 |
| TCGA.QK.A8ZA | alive | 135 | oropharynx | male | 60 | 1 | 1 | 4 |
| TCGA.QK.A8ZB | alive | 135 | larynx | male | 68 | 0 | 1 | 4 |
| TCGA.QK.AA3J | alive | 157 | larynx | male | 69 | 0 | 0 | 1 |
| TCGA.QK.AA3K | alive | 168 | oral tongue | male | 60 | 0 | 1 | 4 |
| TCGA.RS.A6TO | alive | 272 | oral cavity | female | 82 | 0 | 0 | 4 |
| TCGA.RS.A6TP | alive | 236 | tonsil | male | 58 | 1 | 1 | 2 |
| TCGA.T2.A6WX | dead | 209 | oral tongue | female | 73 | 0 | 1 | 3 |
| TCGA.T2.A6WZ | dead | 484 | oral tongue | male | 53 | 0 | 1 | 4 |
| TCGA.T2.A6X0 | alive | 216 | tonsil | male | 49 | 0 | 1 | 4 |
| TCGA.T2.A6X2 | alive | 987 | alveolar ridge | male | 82 | 0 | 1 | 3 |
| TCGA.T3.A92M | alive | 0 | larynx | male | 52 | 0 | 0 | 4 |
| TCGA.T3.A92N | alive | -2 | floor of mouth | male | 79 | 1 | 0 | 4 |
| TCGA.TN.A7HI | alive | 146 | tonsil | male | 56 | 1 | 1 | 1 |
| TCGA.TN.A7HJ | alive | 184 | larynx | male | 51 | 0 | 0 | 3 |
| TCGA.TN.A7HL | alive | 153 | hypopharynx | male | 59 | 1 | 1 | 4 |
| TCGA.UF.A718 | alive | 1326 | larynx | male | 62 | 1 | 0 | 4 |
| TCGA.UF.A719 | alive | 1337 | floor of mouth | male | 54 | 0 | 1 | 3 |
| TCGA.UF.A71A | dead | 86 | floor of mouth | male | 67 | 0 | 0 | 4 |
| TCGA.UF.A71B | alive | 1158 | alveolar ridge | male | 50 | 1 | 1 | 4 |
| TCGA.UF.A71D | alive | 986 | larynx | female | 54 | 1 | 1 | 4 |
| TCGA.UF.A71E | alive | 1090 | floor of mouth | male | 63 | 0 | 1 | 4 |
| TCGA.UF.A7J9 | alive | 1043 | larynx | male | 75 | 1 | 1 | 4 |
| TCGA.UF.A7JA | alive | 1894 | buccal mucosa | female | 66 | 0 | 1 | 4 |
| TCGA.UF.A7JC | alive | 484 | floor of mouth | male | 42 | 1 | 0 | 3 |
| TCGA.UF.A7JD | alive | 653 | buccal mucosa | male | 71 | 1 | 1 | 4 |
| TCGA.UF.A7JF | alive | 1266 | larynx | male | 80 | 1 | 1 | 4 |
| TCGA.UF.A7JH | alive | 469 | larynx | male | 59 | 1 | 1 | 4 |
| TCGA.UF.A7JJ | alive | 214 | larynx | male | 68 | 1 | 0 | 4 |
| TCGA.UF.A7JK | dead | 424 | larynx | male | 59 | 0 | 1 | 4 |
| TCGA.UF.A7JO | dead | 631 | floor of mouth | female | 79 | 0 | 0 | 4 |
| TCGA.UF.A7JS | alive | 413 | oral tongue | male | 59 | 1 | 1 | 4 |
| TCGA.UF.A7JT | dead | 993 | floor of mouth | female | 72 | 0 | 0 | 4 |
| TCGA.UF.A7JV | dead | 90 | hypopharynx | female | 62 | 0 | 0 | 4 |
| TCGA.UP.A6WW | alive | 457 | oral tongue | male | 58 | 0 | 1 | 4 |
| TCGA.WA.A7GZ | alive | 335 | floor of mouth | male | 58 | 1 | 0 | 2 |
| TCGA.WA.A7H4 | alive | 153 | oral tongue | male | 69 | 1 | 0 | 2 |
